# Species composition of sand flies (Diptera: Psychodidae) in caves of Quadrilátero Ferrífero, state of Minas Gerais, Brazil

**DOI:** 10.1371/journal.pone.0220268

**Published:** 2020-03-10

**Authors:** Aldenise Martins Campos, Rodrigo dos Anjos Maia, Débora Capucci, Adriano Pereira Paglia, José Dilermando Andrade Filho

**Affiliations:** 1 Grupo de Estudos em Leishmanioses, Centro de Pesquisas René Rachou, Fundação Oswaldo Cruz, Belo Horizonte, Minas Gerais, Brasil; 2 Departamento de Biologia Geral, Universidade Federal de Minas Gerais, Instituto de Ciências Biológicas, Belo Horizonte, Minas Gerais, Brasil; Universidade Federal da Bahia, BRAZIL

## Abstract

Caves are extreme and inhospitable environments that can harbor several species of vertebrate and invertebrate animals. Among these animals are phlebotomine sand flies, vectors of parasites of the genus *Leishmania* that cause leishmaniasis. This study aimed to evaluate the species composition of sand flies of four caves, a cave located at Moeda Sul (MS) and three at Parque Estadual Serra do Rola Moça (PESRM), in the region of the Quadrilátero Ferrífero in the state of Minas Gerais, southeastern Brazil. Sand flies were collected with automatic light traps. Non-Metric Multidimensional Scaling, using a dissimilarity matrix calculated with the Jaccard index, and Multivariate Permutation Analysis were used to evaluate sand fly species composition among entrance, interior, and the surrounding environments of each sampled cave and to infer biological mechanisms from patterns of distribution among these different cave environments. A total of 375 phlebotomine sand flies representing 14 species and six genera were collected. The most abundant species were *Evandromyia tupynambai* (54.7%), *Brumptomyia troglodytes* (25.6%), *Evandromyia edwardsi* (6.1%), *Psathyromyia brasiliensis* (4.8%) and *Lutzomyia longipalpis* (4.3%). Thirty individuals were collected at MS, 16 inside the cave and 14 from its surroundings. At PESRM, five individuals were collected from the surroundings of cave RM38, 190 individuals from cave RM39 (48 in the cave and 142 from its surroundings) and 150 individuals from cave RM40 (42 in the cave and 108 from its surroundings). The results revealed a rich sand fly fauna with similar species compositions among the entrance, interior, and surrounding environments of each sampled cave, suggesting that both caves and their surroundings are important for maintaining sand fly communities.

## Introduction

Phlebotomines sand flies are insects of the family Psychodidae, subfamily Phlebotominae (Diptera). They are invertebrate hosts for protozoan parasites of the genus *Leishmania*, which cause leishmaniases in humans and other mammals. These insects can be found in urban, rural and wild environments, including caves [1–4].

Cave environments, though seemingly inhospitable due to the lack of light in areas furthest from the cave entrance and the low availability and variety of resources [5, 6], can harbor many species of vertebrates and invertebrates, including phlebotomine sand flies [7–13, 4].

Studies conducted inside caves have produced interesting findings, such as greater, minor or equal diversity of sand flies inside caves compared to their surrounding environments, and differences in the species composition of sand fly communities of caves and their surrounding areas, with species unique to each environment and some common to both [14, 15, 11]. Some species have been described in caves [16, 17, 8, 10], and differences in activity periods and diversity among several caves of the same region have been reported [18, 19, 20]. Studying five caves and their surroundings over two seasons of the year, Campos et al. [4] showed that sand flies exhibit differences in the rhythm of their activity among the interior, entrance and surrounding areas of caves due to differences in the presence of sunlight, which suppresses the flight activity of these mainly nocturnal insects. Despite all these investigations, however, little is known about the distribution patterns of sand flies within caves and in their surroundings even though species composition is known to differ.

Sand fly communities structure between caves and their surroundings have systematically been compared by few studies, namely Galati et al. [14, 15] and Carvalho et al. [11]. Other studies were restricted to caves, either evaluating few months of the year or recording new species. Therefore, no ecological studies have been carried out in ferruginous environments evaluating the environmental gradient of caves and its surrounding phlebotomine communities.

The present study aimed to use statistical analysis to evaluate similarity on sand fly species composition based on species presence or absence along an environmental gradient. This type of analysis is particularly well suited to detect whether the local biological communities are structured by ecological variables or environmental gradients. Dissimilarity measures have a long-standing history and have been widely used to evaluate different biotas distribution [21–23].

Thus, the study evaluated the similarity in species composition of sand flies among the entrance, interior, and surrounding environments of caves at two different locations in the region of the Quadrilátero Ferrífero, state of Minas Gerais. The use of dissimilarity indexes is important for inferring biological mechanisms from sand fly distribution.

## Materials and methods

### Study area

**Fig 1:**
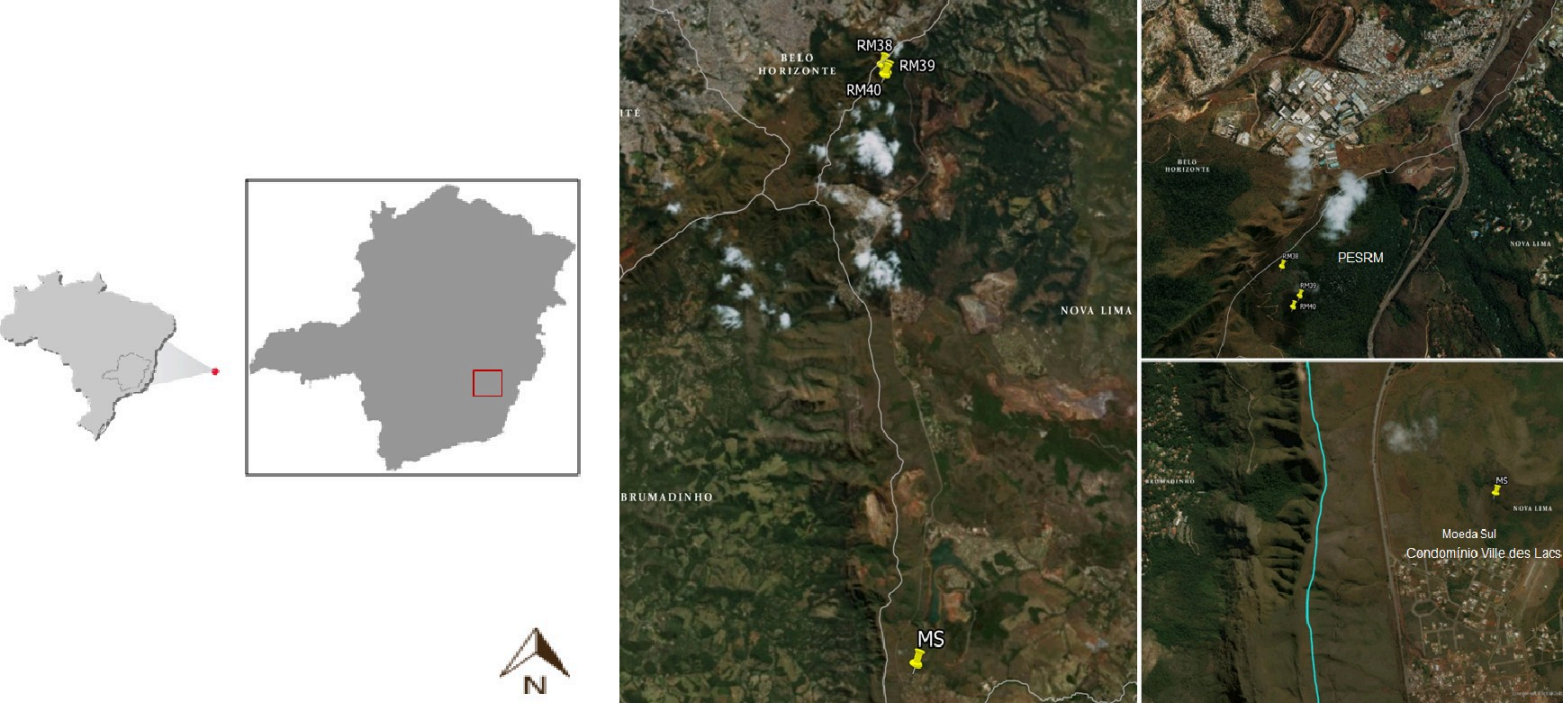
Location of the study area in Moeda Sul (MS) and Parque Estadual Serra do Rola Moça (PESRM), municipality of Nova Lima, MG.

The present study investigated caves and their surroundings at two different locations in the state of Minas Gerais, Brazil: Moeda Sul (MS) and Parque Estadual Serra do Rola Moça (PESRM) (Fig 1). These locations are within the region of the Quadrilátero Ferrífero (Iron Quadrangle) of Minas Gerais, a region with a great number of recorded caves, intense mining activity, real estate exploitation and urbanization [24, 25]. The region possesses abundant ferruginous fields called “cangas”, a rare geological formation found only in the Quadrilátero Ferrífero of Minas Gerais and in Carajás in the state of Pará [26, 27], as well as high altitude fields beyond areas of “capoeira” (secondary formations) [27, 28].

**Fig 2:**
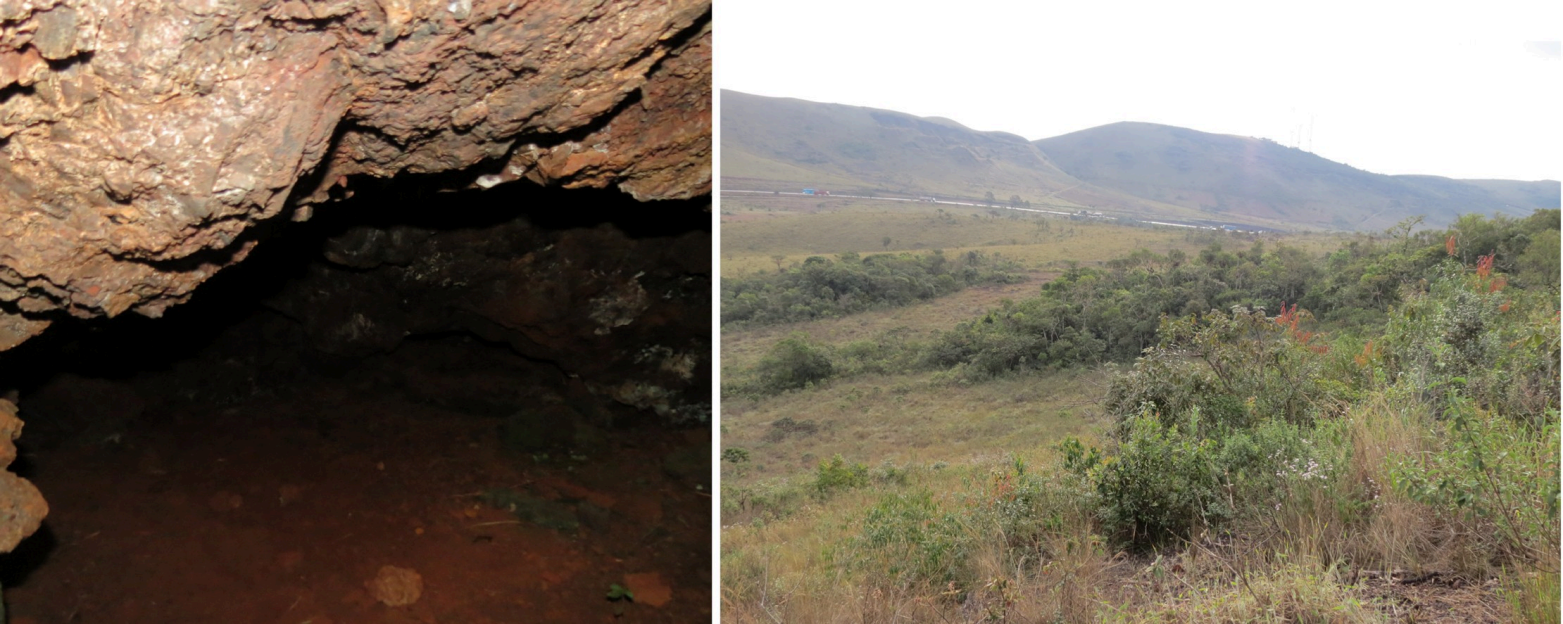
Cave MS and its surroundings in Moeda Sul (MS), Minas Gerais, Brazil.

**Fig 3:**
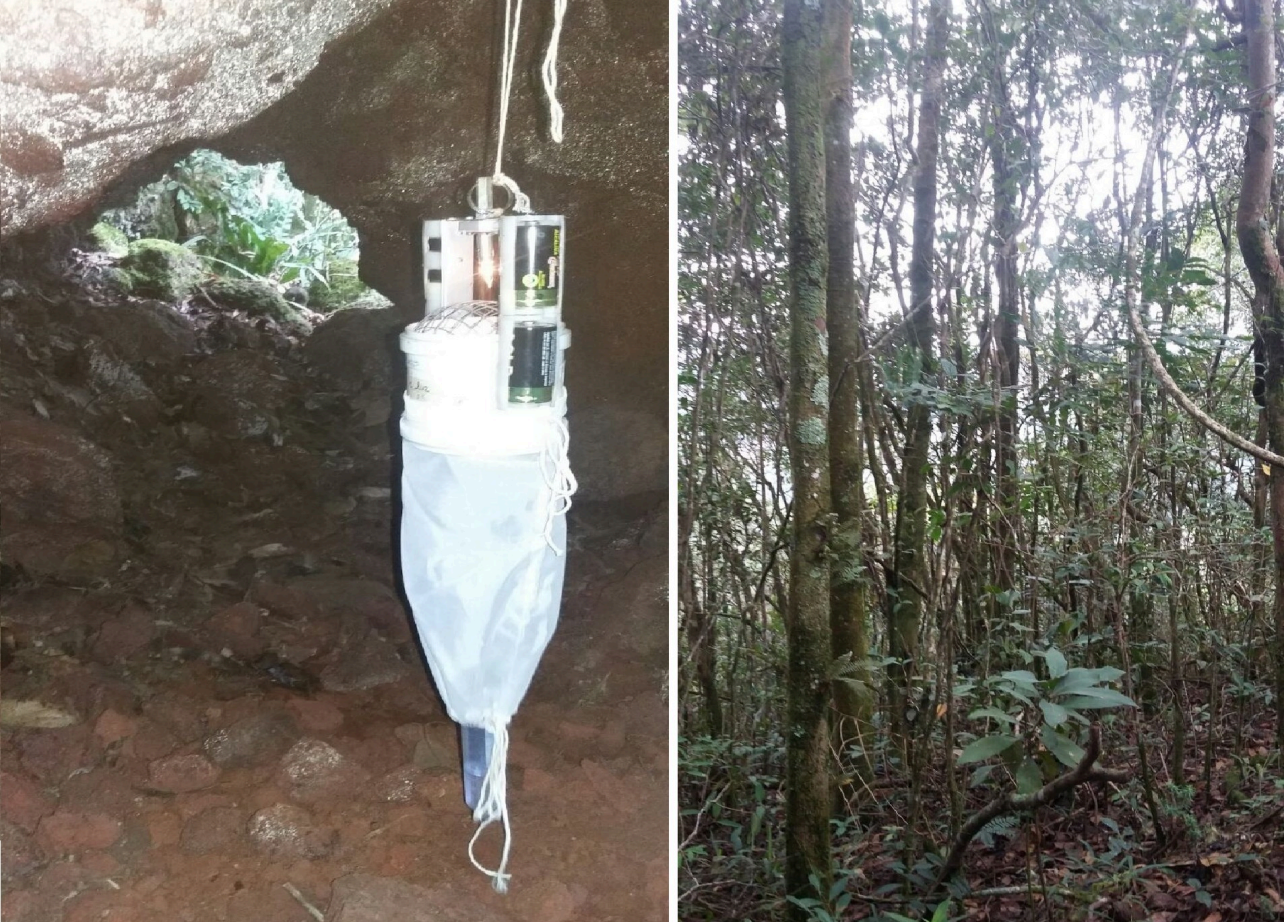
Cave RM38 and its surroundings in Parque Estadual Serra do Rola Moça (PESRM), Minas Gerais, Brazil.

**Fig 4:**
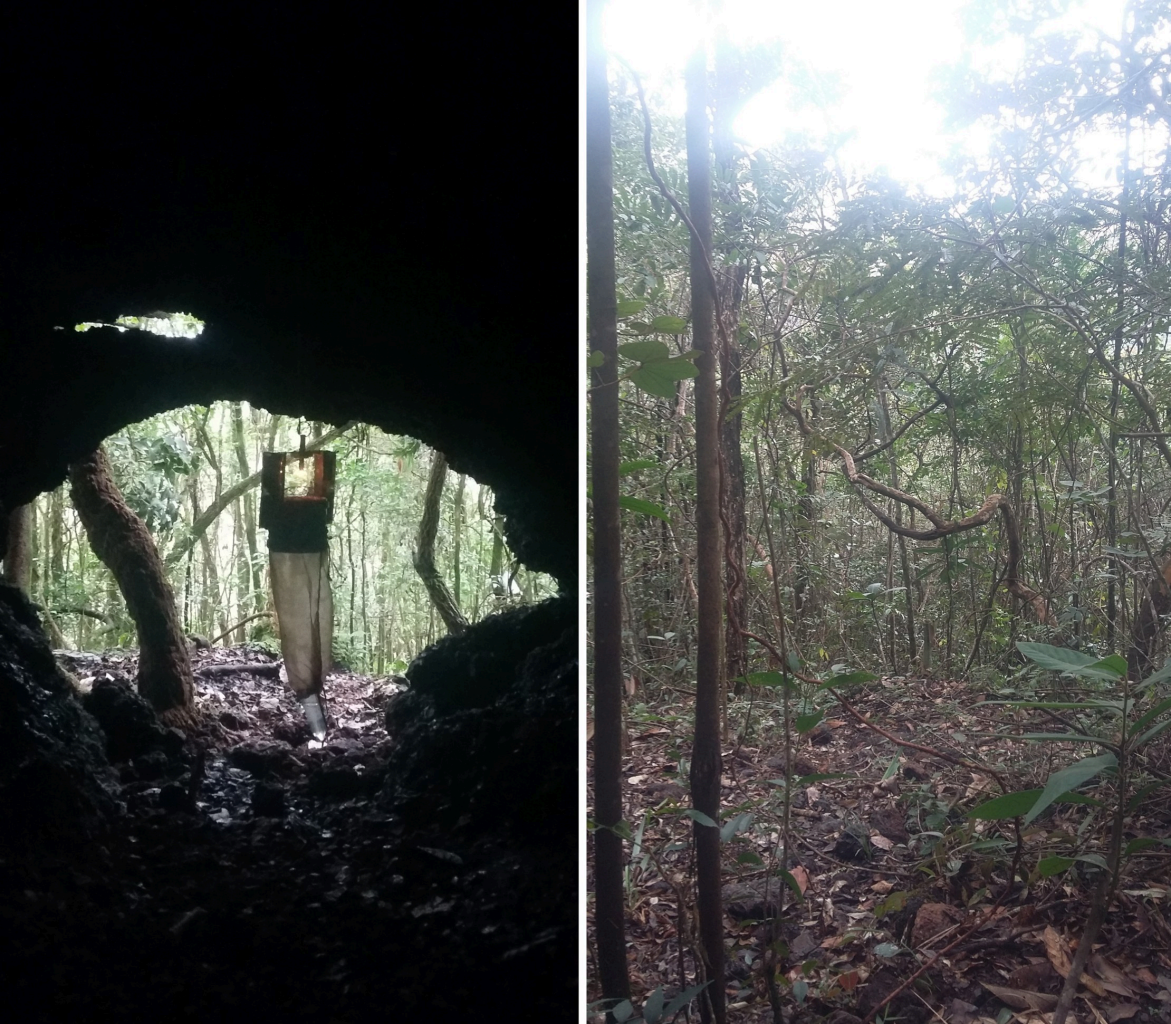
Cave RM39 and its surroundings in Parque Estadual Serra do Rola Moça (PESRM), Minas Gerais, Brazil.

**Fig 5:**
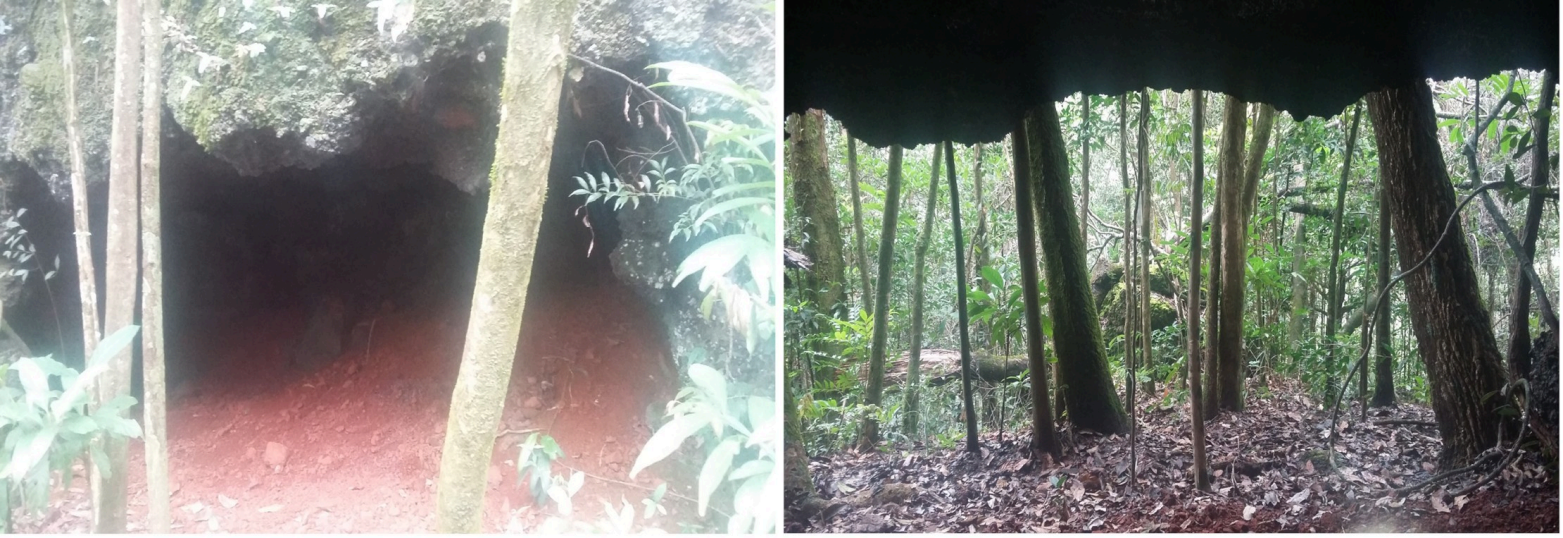
Cave RM40 and its surroundings in Parque Estadual Serra do Rola Moça (PESRM), Minas Gerais, Brazil.

The study focused on four caves and their surroundings, all named by Centro Nacional de Pesquisa e Conservação de Cavernas (CECAV): cave MS at MS, and caves RM38, RM39 and RM40 at PESRM (Figs 2–5 and Table 1). The caves at PESRM are located about 500 m from each other and about 20 km from cave MS. Caves RM39 and RM40 do not have an aphotic zone, whereas caves MS and RM38 have aphotic zone that reaches about 5 m into the interior from the entrance.

**Table 1:** Description of the studied caves and their surroundings at Moeda Sul (MS) and Parque Estadual Serra do Rola Moça (PESRM), municipality of Nova Lima, MG, in the region of the Quadrilátero Ferrífero. Caves were sampled from February 2014 to March 2015.

Description of caves				
Location	Name	Coordinates	Elevation (m a.s.l)	Horizontal extension (m)
MS	MS	20°12'7"S, 43°58'2"W	1,440	70
PESRM	RM38	20°00'48"S, 43°58'43"W	1,363	50
	RM39	20°00'56"S, 43°58'38"W	1,272	30
	RM40	20°00'59"S, 43°58'40"W	1,264	20

All caves located at MS and PESRM are “canga” caves with no running water. They are located in the municipality of Nova Lima in a transition zone between the Cerrado and Atlantic Forest biomes. Cave MS and its surroundings are located in a region of intense exploitation for real estate and urbanization [25], while the caves and their surroundings of PESRM are located in a largely conserved forest area.

### Collection of sand flies

Sand flies were collected under license of Ministério do Meio Ambiente do Brasil (Nº 45636–1) and Instituto Estadual de Florestas de Minas Gerais (Nº 082/2014) (S1 and S2 Files).

Systematic collections were performed using HP-model automatic light traps [29], throughout an entire year: April 2014 to March 2015 at MS, and February 2014 to January 2015 at PESRM. Sampling took place monthly for three consecutive days for a total of 72 h of sampling effort per trap per month.

At MS, four traps were installed in cave MS and eight in its surroundings for a total of 12 traps. At PESRM, three traps were placed in cave RM38 and six traps in its surroundings for a total of nine traps, and two traps were placed in caves RM39 and RM40 and four in their surroundings for a total of six traps each. Thus, a total of 33 traps were used with 11 traps inside of caves and 22 in cave surroundings. For all the caves, the first interior trap was set at the entrance and the others every 10 m into the cave. The first trap in the surroundings was set 10 m from the cave entrance in the forest and the others every 10 m further. All traps were installed approximately 1 m above the ground.

The number of traps installed inside a given cave was the maximum numbered possible based the aforementioned spacing, the horizontal extension of the cave and the difficulty of moving inside. Thus, for a better sampling of the sand fly fauna of the forest fragments surrounding these caves twice the number of interior traps was placed in the surroundings of each cave.

After sampling, the traps were removed and the sand flies killed in glycerinated alcohol, sorted and sexed. Males and females were then placed in test tubes containing 70% alcohol.

### Sand fly identification

Male sand flies were prepared and mounted in Canada Balsam. Female sand flies had their head, thorax and abdomen separated and mounted in Berlese medium, with the head positioned ventrally. All sand flies were identified following the key and classification of Galati [30]. Specimens will be deposited in the Coleção de Flebotomíneos of the Centro de Pesquisas René Rachou/Fiocruz (FIOCRUZ/COLFLEB).

### Statistical analysis

Similarity of species composition among the entrance, interior, and surrounding environments of each sampled cave (caves MS, RM39 and RM40) was determined using Non-Metric Multidimensional Scaling (NMDS) based on a dissimilarity matrix calculated with the Jaccard index (species presence or absence). This method is considered useful for identifying patterns in ecological data. Non-parametric PERMANOVA (Multivariate Permutation Analysis) with 999 replications across the distance matrices [31] was used to test if species composition differed among the environments (entrance, interior, and surroundings) of each sampled cave (MS cave, RM39 cave and RM40 cave). All analyses were performed using R 3.2.4 software [32] (Fig 2).

Analyses by NMDS and Non-parametric PERMANOVA considered one sampling point at the entrance, one in the interior and one in the surroundings of each studied cave. No phlebotomine sand flies were caught inside cave RM38, and so it was removed from the NMDS analysis.

## Results

A total of 375 phlebotomine sand flies of 14 species and six genera were collected. The number of specimens and species in all environments (cave and surroundings) are shown in Table 2. The most abundant species was *Evandromyia tupynambai* (54.7%), followed by *Brumptomyia troglodytes* (25.6%), *Evandromyia edwardsi* (6.1%), *Psathyromyia brasiliensis* (4.8%) and *Lutzomyia longipalpis* (4.3%). All other species accounted for 4.5% of the total (Table 2).

**Table 2:** Phlebotomine sand flies collected in caves and in their respective surroundings in Moeda Sul (MS) and Parque Estadual Serra do Rola Moça (PESRM), municipality of Nova Lima, MG, from February 2014 to March 2015. “S” after the cave name indicates samples collected from cave surroundings; “CAV” indicates samples collected from within the cave.

Species Study locations	Moeda Sul (MS)			Parque Estadual Serra do Rola Moça (PESRM)							TG (%)
Collection environments	MS_S	MS_CAV	TL	RM38_S	RM38_CAV	RM39_S	RM39_CAV	RM40_S	RM40_CAV	TL	
Individuals/environments	TE (%)	TE (%)		TE (%)	TE (%)	TE (%)	TE (%)	TE (%)	TE (%)		
*Brumptomyia troglodytes*	-	-	-	4 (80.0)	-	39 (81.2)	9 (6.3)	36 (85.7)	8 (7.4)	96	96 (25.6)
*Evandromyia edwardsi*	1 (6.2)	-	1	-	-	1 (2.1)	21 (14.8)	-	-	22	23 (6.1)
*Evandromyia evandroi*	-	1 (7.1)	1	-	-	-	-	-	-	-	1 (0.3)
*Evandromyia termitophila*	-	1 (7.1)	1	-	-	-	-	-	-	-	1 (0.3)
*Evandromyia tupynambai*	1 (6.2)	-	1	-	-	3 (6.3)	108 (76.1)	-	93 (86.1)	204	205 (54.7)
*Lutzomyia ischyracantha*	1 (6.2)	-	1	-	-	-	-	-	-	-	1 (0.3)
*Lutzomyia longipalpis*	1 (6.2)	5 (35.7)	6	-	-	-	2 (1.4)	1 (2.4)	7 (6.5)	10	16 (4.3)
*Lutzomyia renei*	-	-	-	1 (20.0)	-	-	-	-	-	1	1 (0.3)
*Nyssomyia whitmani*	-	-	-	-	-	-	-	1 (2.4)	-	1	1 (0.3)
*Psathyromyia brasiliensis*	11 (69.0)	7 (50.0)	18	-	-	-	-	-	-	-	18 (4.8)
*Psathyromyia limai*	-	-	-	-	-	1 (2.1)	-	-	-	1	1 (0.3)
*Pintomyia fischeri*	-	-	-	-	-	-	-	1 (2.4)	-	1	1 (0.3)
*Pintomyia misionensis*	1 (6.2)	-	1	-	-	-	-	-	-	-	1 (0.3)
*Pintomyia monticola*	-	-	-	-	-	4 (8.3)	2 (1.4)	3 (7.1)	-	9	9 (2.4)
Individuals/environments	16 (100.0)	14 (100.0)	30	5 (100.0)	-	48 (100.0)	142 (100.0)	42 (100.0)	108 (100.0)	345	375 (100.0)
Total species	6	4	8	2	0	5	5	5	3	9	14

Environments: MS_S and MS_CAV = Moeda Sul surroundings and cave; RM38_S and RM38_CAV = Rola Moça 38 surroundings and cave; RM39_S and RM39_CAV = Rola Moça 39 surroundings and cave, RM40_S and RM40_CAV = Rola Moça 40 surroundings and cave. TE, TL and TG = total sandflies per environment, location and general.

Thirty individuals were collected at cave MS, with 16 coming from inside the cave and 14 from its surroundings. The most abundant species in cave MS was *Pa*. *brasiliensis* (50%) followed by *Lu*. *longipalpis* (35.7%), whereas the most abundant species in the surroundings was *Pa*. *brasiliensis* (69%). Only five individuals, belonging to *Br*. *troglodytes* (80.0%) and *Lutzomyia renei* (20.0%), were collected from the surroundings of cave RM38 (none from inside the cave). In Cave RM39, a total of 190 individuals were collected: 48 inside and 142 outside. *Ev*. *tupynambai* (76.1%) was the most abundant species inside whereas *Br*. *troglodytes* (81.2%) was the dominant species outside. A total of 150 individuals were collected at cave RM40, with 42 coming from inside the cave and 108 in its surroundings, with *Ev*. *tupynambai* (86.1%) being the most abundant species inside the cave and *Br*. *troglodytes* (85.7%) the most abundant in its surroundings (Table 2).

**Fig 6:**
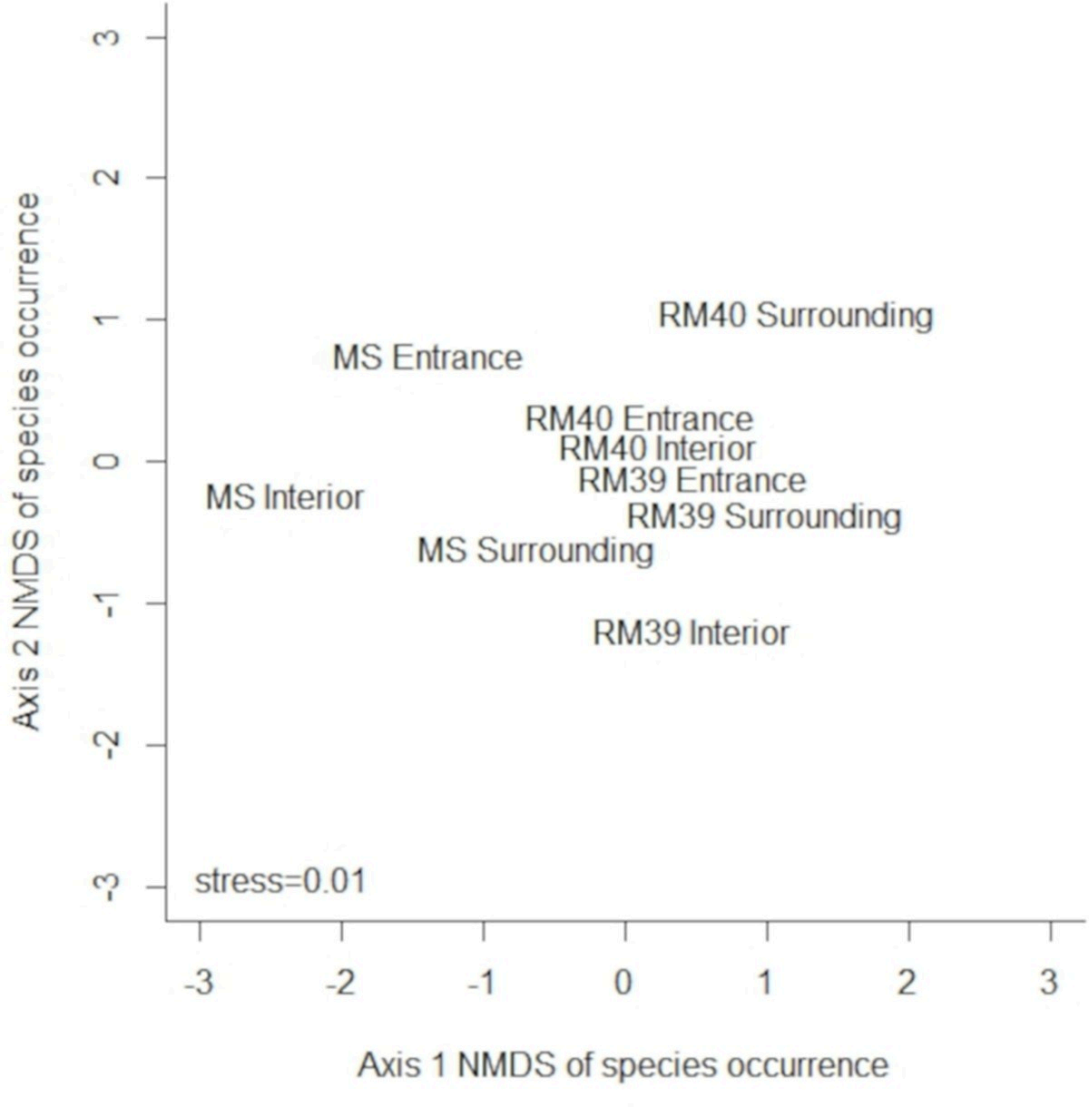
Non-Metric Multidimensional Scaling (NMDS) using a Jaccard dissimilarity matrix calculated with species presence or absence data among the entrance, interior, and surroundings of each sampled cave (caves MS, RM39 and RM40).

Non-Metric Multidimensional Scaling (NMDS), calculated with species presence or absence data, explained 80% of the variation in the data on two axes (Fig 6). The permutation test revealed that sand fly species composition did not differ among entrance, interior, and surroundings of each of the sampled caves (caves MS, RM39 and RM40) (PERMANOVA: *F*_1_ = 0.49; *R*^*2*^ = 0.06; *P* = 0.87).

## Discussion

The results of the present study showed that sand fly species composition did not differ among the entrance, interior, and surroundings of each sampled cave of the locations MS and PESRM, indicating that many species appear to use the caves as places for rest, protection, shelter, breeding, and feeding, as reported by Carvalho et al. [11]. According to Galati et al. [20], sand flies commonly use cave environments for shelter or as breeding sites, or as preferred habitat. The latter was also observed by Alves et al. [18], who recorded the presence of *Deanemyia maruaga*, a species restricted to the interior of caves, in the municipality of Manaus, state of Amazonas. In this study also was observed a low number of sand flies collected, which may be explained by the remarkable severity of the external environment in ferruginous systems [33].

The phlebotomine sand fly fauna inside and outside of the studied caves was found to be very diverse, with some species restricted to caves, others restricted to cave surroundings and others common to both environments. Cave environments with rich sand fly faunas have been reported previously by Carvalho et al. [11] for caves in the municipality of Lassance, Minas Gerais; Campos et al. [4] in the municipality of Pains, Minas Gerais; and Galati et al. [14, 15] for caves in the state of São Paulo.

The most abundant species in our study—*Br*. *troglodytes*, *Ev*. *edwardsi* and *Lu*. *longipalpis*—are commonly found both inside and outside of caves [20, 14, 15, 19, 11, 4]. Other abundant species were *Pa*. *braziliensis* and *Ev*. *tupynambai*, which have few and no records for caves, respectively.

The fact that the caves and their surroundings of PESRM are in a conserved forest fragment can explain the occurrence of *Ev*. *tupynambai* and *Br*. *trogodytes* as the most abundant species inside and outside of the caves, respectively, at that location. These species are very common in conserved forest environments, although they have also been found in shelters for domestic animals, marginal areas and human habitations [34, 14, 35]. Finding *E*. *tupynambai* to be the most abundant species inside the studied caves was a surprise since, despite its wide distribution, there are no records in the literature of this species occurring in cave environments, although a very closely related species, *E*. *petropolitana*, was reported from a stone slot [36]. On the other hand, the finding of *Br*. *trogodytes* as the most abundant species in cave surroundings was expected since it is a characteristic species of forest areas [35].

The cave and its surroundings at MS are situated in a very anthropized region surrounded by a residential area in a periurban environment and a Brazilian federal highway. It was not surprising, therefore, to find the most abundant species to be *Pa*. *brasiliensis* and *Lu*. *longipalpis* since they are very common species in areas of secondary forest, urban areas and domestic animal shelters [37, 3, 4]. There is no record in the literature of *Pa*. *brasiliensis* being the most abundant species inside a cave environment, as was found here, but it being the most abundant in cave surroundings is not unexpected since it is known to occur in anthropized areas [37]. *Lutzomyia longipalpis* was also one of the most abundant species inside cave MS, which corroborates Campos et al. [4], who found this species to be one of the most abundant species inside caves in another area of Minas Gerais.

The species incriminated in the transmission of leishmaniasis to human found in the present study—*Lu*. *longipalpis* and *Nyssomyia whitmani*—have been previously recorded from cave environments by other authors [20, 15, 11, 4]. The other species found are not implicated in transmission of *Leishmania* parasites to human.

The majority of the sand flies collected in the present study were trogloxenes and *Ev*. *tupynambai* and *Ev*. *edwardsi*, the two most abundant species. These two species can be considered troglophiles since they were recorded in almost every month of collection with more than 90% of the individuals occurring inside the cave. This result corroborates Carvalho et al. [11] who reported the presence of trogloxenes and troglophile phlebotomines.

An interesting finding of the present study was the lack of any record of sand flies at the entrance or in the interior of cave RM38 of PESRM, only in its surroundings. One possible explanation is the very small entrance of this cave in relation to the other caves of PESRM. Studying several caves in Serra da Bodoquena, state of Mato Grosso do Sul, Galati et al. [20] found three caves with low densities of sand flies, with the one with the highest density having a wide entrance, which may be more attractive to several species of trogloxenes.

In summary, a rich phlebotomine fauna was recorded for MS and PESRM, with similar sand fly communities among the entrance, interior, and surroundings of each sampled cave. Furthermore, species incriminated in *Leishmania* transmission were recorded, including the presence of *Lu*. *L*. *longipalpis* in the surroundings of cave MS. Even though this species occurred in low abundance at this cave, it deserves special attention because the cave is close to a residential area and it is the main vector of *Leishmania infantum*, the causative agent of visceral leishmaniasis [38]. Therefore, we conclude that both caves and their surroundings are important for the maintenance of sand fly communities. Considering the low frequencies of most species of sand flies and their collection in the surroundings, it seems that these insects are using caves as shelters and resting places for the species that reproduce in the surroundings, which would therefore classify them as trogloxenes. In addition, some species may be practicing hematophagia inside and outside of the caves—because bats and guano were observed inside the caves—and getting carbohydrate energy from around the caves, which would identify them as troglophiles.

In the present study, some limitations were found, such as: at the beginning of the project our idea was to measure humidity and temperature in caves versus their surroundings, however we had several problems with the devices, from thermometer breakage to loss (probable theft), so we did not measure the climate variables; the caves studied were difficult to access, which made a longer collection period impossible and last the *Leishmania* detection study was not performed in the studied sites, because we had methodological problems when performing sample extraction which made this part of the study impossible.

We believe that the limitations mentioned did not compromise the conclusions of study. The proposed purpose was to evaluate the spatial distribution of the species of sand flies at different cave environments and their surroundings, therefore, it does not depend necessarily of seasonality studies, environmental descriptors and molecular biology, although important, to obtain an appropriate and reliable conclusion. For this purpose, we consider the collection of sand flies in different sampled locations to be more appropriate than over a two-year period, since the composition of sand flies from one year to the next varies more in terms of abundance distribution than in terms of presence or absence. A study carried out in an urban environment in the state of Minas Gerais found a similarity in the occurrence of sand flies between collection years, but with variation only in the distribution of abundance of these species (unpublished data). Thus, we consider a collection year as a satisfactory time to assess the presence or absence of sand fly species at a given location.

The low number of sand flies collected is a characteristic of the studied region (Quadrilátero Ferrífero) and given that, we prefer not to add the sand fly number per night and traps, as it would be a very low number. The fact that it has few sand flies compared, for example, to a limestone cave [1, 4] should not be an impediment to studying it. It is important to point out that the low number of sand flies collected may be explained by the high altitude of the studied sites, environmental degradation due to anthropic activities in the study region and the environmental severity of ferruginous grasslands. Despite the low number of sand flies collected over the one-year period, the statistical analysis chosen for the data were adequate for drawing reliable conclusions, since it was based on presence or absence species data in the community (not species abundance), which was very diverse.

## Supporting information

S1 File License of Ministério do Meio Ambiente do Brasil. (PDF)

S2 File License of Instituto Estadual de Florestas de Minas Gerais. (DOC)
