# Supplementary material for: Species composition of sand flies (Diptera: Psychodidae) in caves of Quadrilátero Ferrífero, state of Minas Gerais, Brazil
Source: PLoS One. 2020 Mar 10;15(3):e0220268. doi: 10.1371/journal.pone.0220268 (PMC7064241; doi:10.1371/journal.pone.0220268)
Supplement: S1 File — (PDF) [file pone.0220268.s001.pdf]

Ministério do Meio Ambiente - MMA
Instituto Chico Mendes de Conservação da Biodiversidade - ICMBio
Sistema de Autorização e Informação em Biodiversidade - SISBIO

Autorização para atividades com finalidade científica

Número: 45636-1	Data da Emissão: 06/10/2014 13:31	Data para Revalidação*: 05/11/2015
* De acordo com o art. 33 da IN 154/2009, esta autorização tem prazo de validade equivalente ao previsto no cronograma de atividades do projeto, mas deverá ser revalidada anualmente mediante a apresentação do relatório de atividades a ser enviado por meio do Sisbio no prazo de até 30 dias a contar da data do aniversário de sua emissão.		

Dados do titular

SISBIO

Nome: ALDENISE MARTINS CAMPOS	CPF: 672.188.573-53
Título do Projeto: Estudos ecopidemiológicos dos hospedeiros vertebrados e invertebrados de Leishmania ssp. em ambientes cavernícolas do Quadrilátero Ferrífero e da região cárstica de Pains, Minas Gerais, Brasil	
Nome da Instituição : UFMG - UNIVERSIDADE FEDERAL DE MINAS GERAIS	CNPJ: 17.217.985/0001-04

Cronograma de atividades

#	Descrição da atividade	Início (mês/ano)	Fim (mês/ano)
1	Captura e coleta das amostras biológicas dos pequenos mamíferos	09/2014	07/2015
2	Coleta de Flebotomíneos	09/2014	08/2015
3	Investigação da infecção natural em flebotomíneos e pequenos mamíferos	09/2014	10/2015
4	Identificação dos flebotomíneos e Pequenos mamíferos	09/2014	10/2015
5	Investigação da fonte alimentar sanguínea em fêmeas de flebotomíneos	09/2014	12/2015
6	Análise dos dados	12/2015	02/2016
7	Redação de artigo e relatórios	12/2015	08/2017

Observações e ressalvas

1	As atividades de campo exercidas por pessoa natural ou jurídica estrangeira, em todo o território nacional, que impliquem o deslocamento de recursos humanos e materiais, tendo por objeto coletar dados, materiais, espécimes biológicos e minerais, peças integrantes da cultura nativa e cultura popular, presente e passada, obtidos por meio de recursos e técnicas que se destinem ao estudo, à difusão ou à pesquisa, estão sujeitas a autorização do Ministério de Ciência e Tecnologia.
2	Esta autorização NÃO exime o pesquisador titular e os membros de sua equipe da necessidade de obter as anuências previstas em outros instrumentos legais, bem como do consentimento do responsável pela área, pública ou privada, onde será realizada a atividade, inclusive do órgão gestor de terra indígena (FUNAI), da unidade de conservação estadual, distrital ou municipal, ou do proprietário, arrendatário, posseiro ou morador de área dentro dos limites de unidade de conservação federal cujo processo de regularização fundiária encontra-se em curso.
3	Este documento somente poderá ser utilizado para os fins previstos na Instrução Normativa IBAMA n° 154/2007 ou na Instrução Normativa ICMBio n° 10/2010, no que especifica esta Autorização, não podendo ser utilizado para fins comerciais, industriais ou esportivos. O material biológico coletado deverá ser utilizado para atividades científicas ou didáticas no âmbito do ensino superior.
4	A autorização para envio ao exterior de material biológico não consignado deverá ser requerida por meio do endereço eletrônico www.ibama.gov.br (Serviços on-line - Licença para importação ou exportação de flora e fauna - CITES e não CITES).
5	O titular de licença ou autorização e os membros da sua equipe deverão optar por métodos de coleta e instrumentos de captura direcionados, sempre que possível, ao grupo taxonômico de interesse, evitando a morte ou dano significativo a outros grupos; e empregar esforço de coleta ou captura que não comprometa a viabilidade de populações do grupo taxonômico de interesse em condição in situ.
6	O titular de autorização ou de licença permanente, assim como os membros de sua equipe, quando da violação da legislação vigente, ou quando da inadequação, omissão ou falsa descrição de informações relevantes que subsidiaram a expedição do ato, poderá, mediante decisão motivada, ter a autorização ou licença suspensa ou revogada pelo ICMBio e o material biológico coletado apreendido nos termos da legislação brasileira em vigor.
7	Este documento não dispensa o cumprimento da legislação que dispõe sobre acesso a componente do patrimônio genético existente no território nacional, na plataforma continental e na zona econômica exclusiva, ou ao conhecimento tradicional associado ao patrimônio genético, para fins de pesquisa científica, bioprospecção e desenvolvimento tecnológico. Veja maiores informações em www.mma.gov.br/cgen.
8	Em caso de pesquisa em UNIDADE DE CONSERVAÇÃO, o pesquisador titular desta autorização deverá contactar a administração da unidade a fim de CONFIRMAR AS DATAS das expedições, as condições para realização das coletas e de uso da infra-estrutura da unidade.

Outras ressalvas

1	Não está autorizada a coleta, transporte de fêmeas grávidas ou em processo de amamentação.<br>As armadilhas utilizadas para a captura de pequenos mamíferos deverão ser vistoriadas pelo menos duas vezes ao dia (pela manhã e tarde) para minimizar a morte devido a hipo ou hipertermia.<br>As redes de neblinas deverão ser verificadas no mínimo de 30 em 30 minutos.
2	A pesquisadora deverá enviar ao Cecav as coordenadas das cavernas, com a atenção para a coleta ser realizada na maior proximidade de sua entrada principal e na maior precisão possível, atentando para o registro do datum utilizado, do número de satélites e erro associados aos pontos coletados.

Este documento (Autorização para atividades com finalidade científica) foi expedido com base na Instrução Normativa nº154/2007. Através do código de autenticação abaixo, qualquer cidadão poderá verificar a autenticidade ou regularidade deste documento, por meio da página do Sisbio/ICMBio na Internet (www.icmbio.gov.br/sisbio).

SISBIO

Código de autenticação: 65726225

Autorização para atividades com finalidade científica

Número: 45636-1	Data da Emissão: 06/10/2014 13:31	Data para Revalidação*: 05/11/2015

* De acordo com o art. 33 da IN 154/2009, esta autorização tem prazo de validade equivalente ao previsto no cronograma de atividades do projeto, mas deverá ser revalidada anualmente mediante a apresentação do relatório de atividades a ser enviado por meio do Sisbio no prazo de até 30 dias a contar da data do aniversário de sua emissão.

Dados do titular

Nome: ALDENISE MARTINS CAMPOS	CPF: 672.188.573-53
Título do Projeto: Estudos ecopidemiológicos dos hospedeiros vertebrados e invertebrados de Leishmania ssp. em ambientes cavernícolas do Quadrilátero Ferrífero e da região cárstica de Pains, Minas Gerais, Brasil	
Nome da Instituição : UFMG - UNIVERSIDADE FEDERAL DE MINAS GERAIS	CNPJ: 17.217.985/0001-04

Equipe

#	Nome	Função	CPF	Doc. Identidade	Nacionalidade
1	Rodrigo dos Anjos Maia	colaborador	120.268.336-30	mg14703886 ssp-MG	Brasileira
2	Rafaela Velloso Missagia	Colaborador	096.359.736-16	MG-15.421.450 SSP-MG	Brasileira

Locais onde as atividades de campo serão executadas

#	Município	UF	Descrição do local	Tipo
1	NOVA LIMA	MG	Parque Estadual Serra do Rola Moça	Fora de UC Federal
2	OURO PRETO	MG	Parque Estadual Serra do Ouro Branco	Fora de UC Federal
3	OURO BRANCO	MG	Parque Estadual Serra do Ouro Branco	Fora de UC Federal
4	PAINS	MG	Pains	Fora de UC Federal
5	NOVA LIMA	MG	Área Moeda Sul	Fora de UC Federal

Atividades X Táxons

#	Atividade	Táxons
1	Coleta/transporte de amostras biológicas in situ	Chiroptera, Didelphimorphia, Rodentia
2	Coleta/transporte de espécimes da fauna silvestre in situ	Rodentia (*Qtde: 5), Chiroptera (*Qtde: 5), Didelphimorphia (*Qtde: 5), Psychodidae (*Qtde: 500)

* Quantidade de indivíduos por espécie, por localidade ou unidade de conservação, a serem coletados durante um ano.

Material e métodos

1	Amostras biológicas (Outros mamíferos)	Sangue, Fragmento de tecido/órgão
2	Método de captura/coleta (Invertebrados Terrestres)	Armadilha luminosa
3	Método de captura/coleta (Outros mamíferos)	Rede de neblina, Armadilha tipo gaiola com atração por iscas ("Box Trap/Tomahawk/Sherman")

Destino do material biológico coletado

#	Nome local destino	Tipo Destino
1	CENTRO DE PESQUISAS RENÉ RACHOU-FIOCRUZ	coleção
2	UFMG - UNIVERSIDADE FEDERAL DE MINAS GERAIS	coleção

Este documento (Autorização para atividades com finalidade científica) foi expedido com base na Instrução Normativa nº154/2007. Através do código de autenticação abaixo, qualquer cidadão poderá verificar a autenticidade ou regularidade deste documento, por meio da página do Sisbio/ICMBio na Internet (www.icmbio.gov.br/sisbio).

Código de autenticação: 65726225

Autorização para atividades com finalidade científica

Número: 45636-1	Data da Emissão: 06/10/2014 13:31	Data para Revalidação*: 05/11/2015

* De acordo com o art. 33 da IN 154/2009, esta autorização tem prazo de validade equivalente ao previsto no cronograma de atividades do projeto, mas deverá ser revalidada anualmente mediante a apresentação do relatório de atividades a ser enviado por meio do Sisbio no prazo de até 30 dias a contar da data do aniversário de sua emissão.

Dados do titular

Nome: ALDENISE MARTINS CAMPOS	CPF: 672.188.573-53
Título do Projeto: Estudos ecopidemiológicos dos hospedeiros vertebrados e invertebrados de Leishmania ssp. em ambientes cavernícolas do Quadrilátero Ferrífero e da região cárstica de Pains, Minas Gerais, Brasil	
Nome da Instituição : UFMG - UNIVERSIDADE FEDERAL DE MINAS GERAIS	CNPJ: 17.217.985/0001-04

SISBIO

Registro de coleta imprevista de material biológico

De acordo com a Instrução Normativa nº154/2007, a coleta imprevista de material biológico ou de substrato não contemplado na autorização ou na licença permanente deverá ser anotada na mesma, em campo específico, por ocasião da coleta, devendo esta coleta imprevista ser comunicada por meio do relatório de atividades. O transporte do material biológico ou do substrato deverá ser acompanhado da autorização ou da licença permanente com a devida anotação. O material biológico coletado de forma imprevista, deverá ser destinado à instituição científica e, depositado, preferencialmente, em coleção biológica científica registrada no Cadastro Nacional de Coleções Biológicas (CCBIO).

Táxon*	Qtde.	Tipo de amostra	Qtde.	Data

Este documento (Autorização para atividades com finalidade científica) foi expedido com base na Instrução Normativa nº154/2007. Através do código de autenticação abaixo, qualquer cidadão poderá verificar a autenticidade ou regularidade deste documento, por meio da página do Sisbio/ICMBio na Internet (www.icmbio.gov.br/sisbio).

SISBIO

Código de autenticação: 65726225

Ministério do Meio Ambiente - MMA
Instituto Chico Mendes de Conservação da Biodiversidade - ICMBio
Sistema de Autorização e Informação em Biodiversidade - SISBIO

Autorização para atividades com finalidade científica

Número: 45636-1	Data da Emissão: 06/10/2014 13:31	Data para Revalidação*: 05/11/2015
* De acordo com o art. 33 da IN 154/2009, esta autorização tem prazo de validade equivalente ao previsto no cronograma de atividades do projeto, mas deverá ser revalidada anualmente mediante a apresentação do relatório de atividades a ser enviado por meio do Sisbio no prazo de até 30 dias a contar da data do aniversário de sua emissão.		

Dados do titular

Nome: ALDENISE MARTINS CAMPOS	CPF: 672.188.573-53
Título do Projeto: Estudos ecopidemiológicos dos hospedeiros vertebrados e invertebrados de Leishmania ssp. em ambientes cavernícolas do Quadrilátero Ferrífero e da região cárstica de Pains, Minas Gerais, Brasil	
Nome da Instituição : UFMG - UNIVERSIDADE FEDERAL DE MINAS GERAIS	CNPJ: 17.217.985/0001-04

SISBIO

* Identificar o espécime no nível taxonômico possível.

Este documento (Autorização para atividades com finalidade científica) foi expedido com base na Instrução Normativa nº154/2007. Através do código de autenticação abaixo, qualquer cidadão poderá verificar a autenticidade ou regularidade deste documento, por meio da página do Sisbio/ICMBio na Internet (www.icmbio.gov.br/sisbio).

SISBIO

Código de autenticação: 65726225
