# Supplementary figures and images for: Species composition of sand flies (Diptera: Psychodidae) in caves of Quadrilátero Ferrífero, state of Minas Gerais, Brazil
Source: PLoS One. 2020 Mar 10;15(3):e0220268. doi: 10.1371/journal.pone.0220268 (PMC7064241; doi:10.1371/journal.pone.0220268)

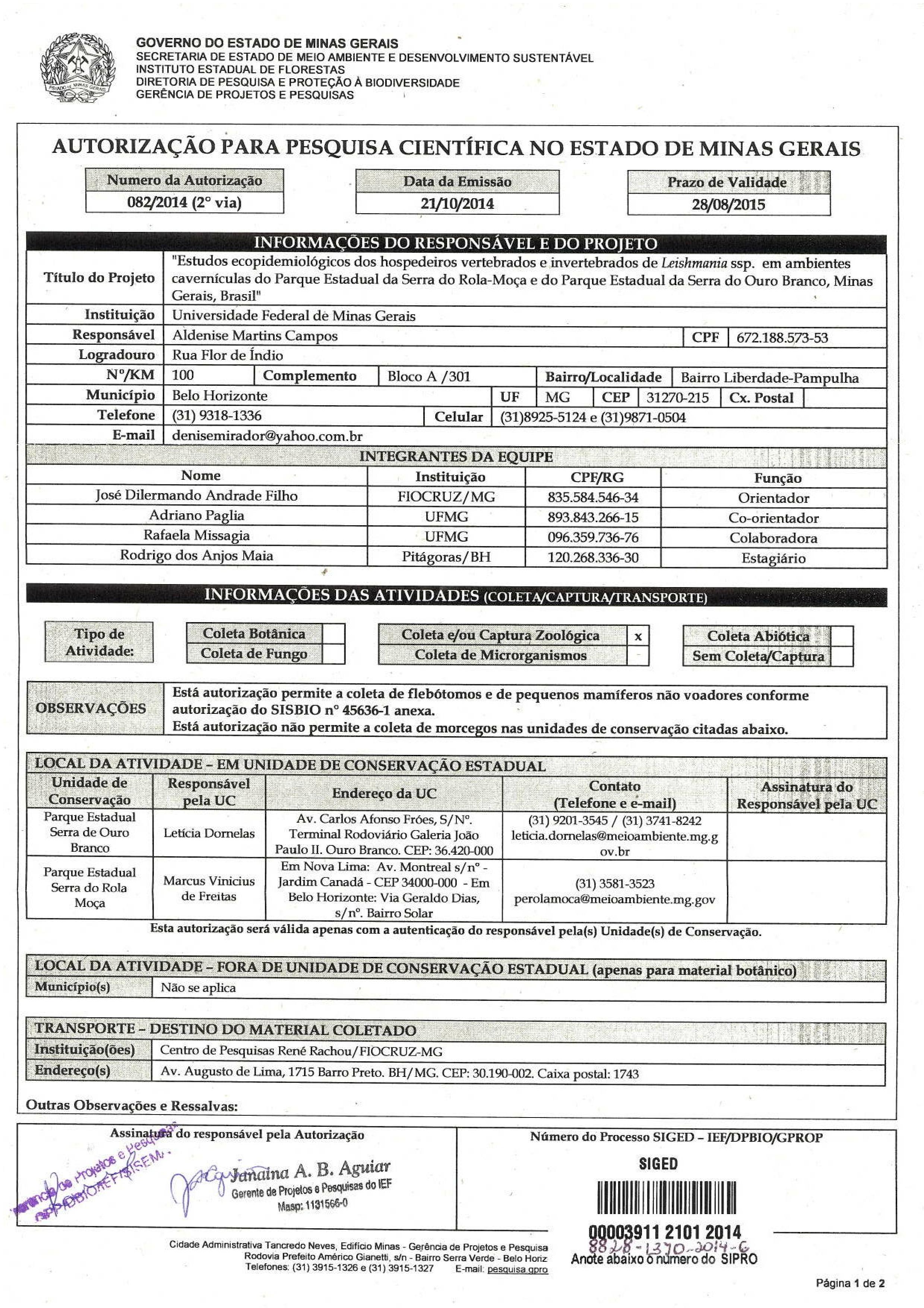


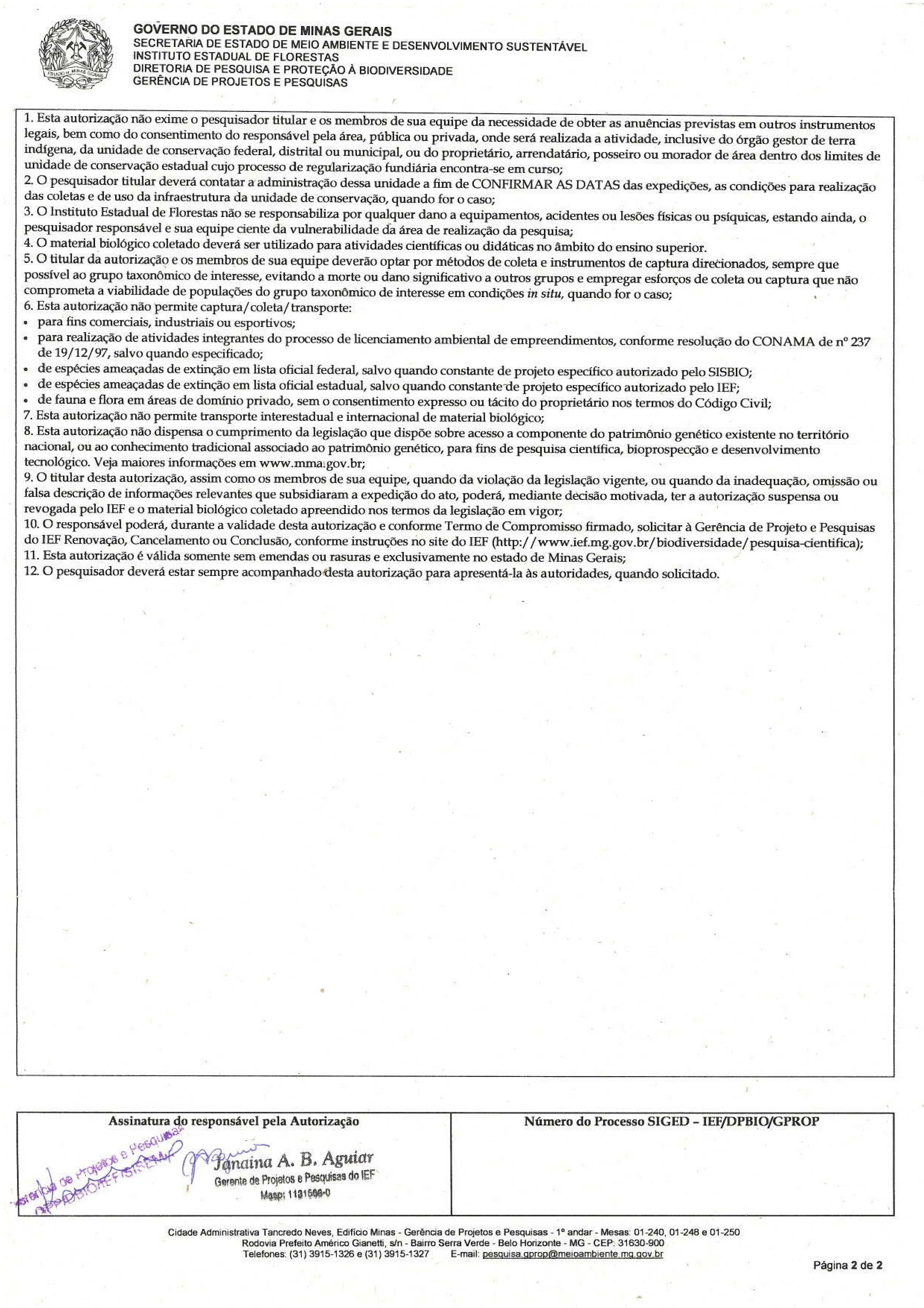

Supplement: S2 File — (DOC) [file pone.0220268.s002.doc]
