# Supplementary material for: Species composition of sand flies (Diptera: Psychodidae) in caves of Quadrilátero Ferrífero, state of Minas Gerais, Brazil
Source: PLoS One. 2020 Mar 10;15(3):e0220268. doi: 10.1371/journal.pone.0220268 (PMC7064241; doi:10.1371/journal.pone.0220268)
Supplement: Attachment — Submitted filename: Response to Reviewers.doc [file pone.0220268.s003.doc]

**Dear editor,**

**Below are the responses to the reviewers' comments.**

**We are available for clarification.**

**Reviewer #1: General comment: Please ask for English revision by a native speaker, the quality of English is not sufficient.**

Answer: The English was revision by a native speaker.

**Abstract: Number of caves should be mentioned.**

Answer: The number of caves was mentioned.

**Introduction: Page1: Reference 1 is obsolete and looks like a Brazilian book which is hardly available in other countries. Consider to replace it by some current review or leave this general statement without any reference.**

Answer: The reference 1 was taken from the page 1 of the introduction.

**Page2: I think that nine references cited for a single simple statement are too much. Is there any review to be referred instead?**

Answer: Only one reference was left for each environment studied, urban, rural, wild and cave (1, 2, 3, 4). And there is no any review to be referred instead.

**15 references mentioned (numbers 2-16) deal with sand fly fauna in caves and this fact puts in doubt the following statement "However, despite all these investigation, little is known of the patterns of distribution of sand flies in caves..." Try to explain more clearly what is the novelty of your work.**

Answer: It appears that many projects have been carried out in Brazil's caves, but given the size of the country and the number of existing caves, this number becomes small. In addition, this is the first systematic work carried out in Brazil's iron caves.

**Materials and Methods: Explain why different numbers of traps were used in different cages and what was the distance between traps within one cage. All these details may affect catches per trap.**

Answer: The caves sizes were different. Was installed the maximum possible number of traps that fit within each cave, however were made some modifications in text to improve understanding. **The distance between traps within one cave is in the text** “For all caves and their surroundings sampled, the first trap inside a cave was at its entrance and the other was set every 10 m further into the cave. In the surroundings, the first trap was set in the forest 10 m from the cave entrance and the others were set every 10 m further away from the cave into the forest.” Modifications were done in the topics Collection of sand flies, Sand fly identification and Statistical analysis of the Materials and Methods.

**Add which keys were used for morphological identification of sand flies.**

Answer: This information was added to the text with some modifications.

**Are there any males or rest of female bodies available in ethanol for eventual molecular studies (confirmation of species identification or population studies) in the future?**

Answer: No. The information in line 152-153 “and females were placed in test tubes containing 6% DMSO for future study of *Leishmania* DNA detection” was taken from the text due to a misunderstanding, since the study was only ecological. In the future we will do studies of this kind.

**Results: Did you trap any males with noninverted genitalia or do you have any other evidence that caves serve as breeding sites for sand flies?**

Answer: Was not found any males with noninverted genitalia, as it is very difficult to collect sand flies at this stage of development. An evidence that caves serve as breeding sites for sand flies was the records of presence of troglophils species.

**In Table 2, explain what is T. Do you mean "n"?**

Answer: Was explain in table caption 2 what T means. We also added other information to table 2.

**I am missing the information about sand fly "density", for example the sand fly number per night and trap.**

Answer: The number of sand flies collected was low, a fact common in this type of environment (Quadrilátero Ferrífero), so we prefer not to do these analyzes of sand flies collected by trap and per night.

**Do you have any data about temperature and humidity in caves versus their surroundings?**

Answer: We do not have this information. At the beginning of the project our idea was to measure humidity and temperature, however we had several problems with the devices, from thermometer breakage to loss (probable theft), so we did not measure the climate variables.

**Discussion
You are frequently using term "condominium". Do you mean village or single house?**
Answer: Condominiums are houses in a periurban environment, surrounded by walls. The entry of people is controlled. It is common in the metropolitan region of Belo Horizonte and presents preserved vegetation and wildlife present near the residents.

**I suggest to improve/change discussion why caves are important for "the maintenance of their sand fly communities". Could you specify if these caves are serving as resting sites, breeding sites, and/or what else?**

Answer: Information was added in this part of the discussion to improve understanding of the conclusions reached.

**Reviewer #2:** The manuscript entitled “Similarity of species composition of sand flies (Diptera: Psychodidae) in caves of Quadrilátero Ferrífero, state Minas Gerais, Brasil“ described an interesting study concerning the sand fly fauna in caves and its surroundings. Several major points need to be addressed before publishing this work, and therefore, this manuscript cannot be published in its actual form.

The authors reported a similarity of sand fly communities among the entrance, interior, and surroundings environments of each sampled cave at places, MS and PESRM. My major concern with this study is that only the analysis was based on a total of 375 sand flies caught during a whole year period. With such low number, no conclusion can be drawn. By using other traps, the authors may increase the number of sand flies needed for an objective statistical analysis. On the other hand, the authors should compare the similarity on a monthly basis and NOT during the whole period because it is well known that phenology differ among species. In addition, the authors should report *Leishmania* infection in both sites.

Answer: The low number of sand flies collected is a characteristic of the studied region (Quadrilátero Ferrífero) and is not a problem of the methodology used. The fact that it has few sand flies compared to, for example, a limestone cave should not be an impediment to studying it. Information was added in the discussion topic for a better understanding of the overall conclusions obtained as well as for the clarification of the statistical analysis used to arrive at an appropriate and reliable conclusion. The comparison of sand flies fauna on a monthly basis will not be possible, since there have been many months with only one species or few species of sand flies collected, which made it impossible to compare the similarity monthly. The NMDS analysis can be considered useful in trying to find out if there are patterns in the ecological data, which was the purpose of the study. Thus, we believe that analyzing the similarity of the phlebotomine community by caves and their surroundings during the entire collection period is the best way for what was proposed in the present study, since the NMDS analysis was to investigate the composition of sand fly species based on the presence and absence of species inside and outside the caves regardless of the sampling period, in other words, was an analysis to evaluate the spatial distribution of the community and not temporal distribution. The *Leishmania* detection study was not performed in the studied sites, because we had methodological problems when performing sample extraction which made this part of the study impossible.
