# Supplementary material for: Species composition of sand flies (Diptera: Psychodidae) in caves of Quadrilátero Ferrífero, state of Minas Gerais, Brazil
Source: PLoS One. 2020 Mar 10;15(3):e0220268. doi: 10.1371/journal.pone.0220268 (PMC7064241; doi:10.1371/journal.pone.0220268)
Supplement: Attachment — Submitted filename: Rebuttal Letter.doc [file pone.0220268.s005.doc]

**Dear Academic Editor,**

**Below the answers to the questions**

1- As repeatedly requested by reviewer 1, please, completely disclose the missing the information about keys used for morphological identification.

Answer: In the last version, the phrase on identification and classification of sandflies used in the article was modified (All sand flies were identified following the key and classification of Galati (2003) [30], lines 140 and 141. In case it is not clear we can change in order to facilitate the understanding of readers

2- Lines 64 to 66. Correct to "Therefore, no ecological studies have been carried out in ferruginous environments evaluating the environmental gradient of caves and its surrounding phlebotomine communities.

Answer: Change accepted

3- Line 70. Correct from "structure" to "structured".

Answer: Change accepted

4- Line 293. add a 'to' in 'for example, to a limestone cave [1, 4]'.

Answer: Change accepted
