# Supplementary material for: Species composition of sand flies (Diptera: Psychodidae) in caves of Quadrilátero Ferrífero, state of Minas Gerais, Brazil
Source: PLoS One. 2020 Mar 10;15(3):e0220268. doi: 10.1371/journal.pone.0220268 (PMC7064241; doi:10.1371/journal.pone.0220268)
Supplement: Peer review history

# Peer review: Decision Letter 0

**DOI:** 10.1371/journal.pone.0220268.r001

**Authors:** Nagila Francinete Costa Secundino

27 Aug 2019

PONE-D-19-18993

Similarity of species composition of sand flies (Diptera: Psychodidae) in caves of Quadrilátero Ferrífero, state Minas Gerais, Brasil

PLOS ONE

Dear Dr. Andrade Filho,

Thank you for submitting your manuscript to PLOS ONE. After careful consideration, we feel that it has merit but does not fully meet PLOS ONE’s publication criteria as it currently stands. Therefore, we invite you to submit a revised version of the manuscript that addresses the points raised during the review process.

We would appreciate receiving your revised manuscript by Oct 11 2019 11:59PM. When you are ready to submit your revision, log on to https://www.editorialmanager.com/pone/ and select the 'Submissions Needing Revision' folder to locate your manuscript file.

If you would like to make changes to your financial disclosure, please include your updated statement in your cover letter.

To enhance the reproducibility of your results, we recommend that if applicable you deposit your laboratory protocols in protocols.io, where a protocol can be assigned its own identifier (DOI) such that it can be cited independently in the future. For instructions see: http://journals.plos.org/plosone/s/submission-guidelines#loc-laboratory-protocols

Please include the following items when submitting your revised manuscript:

- A rebuttal letter that responds to each point raised by the academic editor and reviewer(s). This letter should be uploaded as separate file and labeled 'Response to Reviewers'.
- A marked-up copy of your manuscript that highlights changes made to the original version. This file should be uploaded as separate file and labeled 'Revised Manuscript with Track Changes'.
- An unmarked version of your revised paper without tracked changes. This file should be uploaded as separate file and labeled 'Manuscript'.

Please note while forming your response, if your article is accepted, you may have the opportunity to make the peer review history publicly available. The record will include editor decision letters (with reviews) and your responses to reviewer comments. If eligible, we will contact you to opt in or out.

We look forward to receiving your revised manuscript.

Kind regards,

Nagila Francinete Costa Secundino, Ph.D

Academic Editor

PLOS ONE

Journal Requirements:

1. When submitting your revision, we need you to address these additional requirements.

Please ensure that your manuscript meets PLOS ONE's style requirements, including those for file naming. The PLOS ONE style templates can be found at

http://www.journals.plos.org/plosone/s/file?id=wjVg/PLOSOne_formatting_sample_main_body.pdf and http://www.journals.plos.org/plosone/s/file?id=ba62/PLOSOne_formatting_sample_title_authors_affiliations.pdf

2.  Please include captions for your Supporting Information files at the end of your manuscript, and update any in-text citations to match accordingly. Please see our Supporting Information guidelines for more information: http://journals.plos.org/plosone/s/supporting-information.

[Note: HTML markup is below. Please do not edit.]

Reviewers' comments:

Reviewer's Responses to Questions

**Comments to the Author**

1. Is the manuscript technically sound, and do the data support the conclusions?

The manuscript must describe a technically sound piece of scientific research with data that supports the conclusions. Experiments must have been conducted rigorously, with appropriate controls, replication, and sample sizes. The conclusions must be drawn appropriately based on the data presented. 

Reviewer #1: Partly

Reviewer #2: Partly

**********

2. Has the statistical analysis been performed appropriately and rigorously? 

Reviewer #1: I Don't Know

Reviewer #2: Yes

**********

3. Have the authors made all data underlying the findings in their manuscript fully available?

The PLOS Data policy requires authors to make all data underlying the findings described in their manuscript fully available without restriction, with rare exception (please refer to the Data Availability Statement in the manuscript PDF file). The data should be provided as part of the manuscript or its supporting information, or deposited to a public repository. For example, in addition to summary statistics, the data points behind means, medians and variance measures should be available. If there are restrictions on publicly sharing data—e.g. participant privacy or use of data from a third party—those must be specified.

Reviewer #1: Yes

Reviewer #2: No

**********

4. Is the manuscript presented in an intelligible fashion and written in standard English?

PLOS ONE does not copyedit accepted manuscripts, so the language in submitted articles must be clear, correct, and unambiguous. Any typographical or grammatical errors should be corrected at revision, so please note any specific errors here.

Reviewer #1: No

Reviewer #2: No

**********

5. Review Comments to the Author

Please use the space provided to explain your answers to the questions above. You may also include additional comments for the author, including concerns about dual publication, research ethics, or publication ethics. (Please upload your review as an attachment if it exceeds 20,000 characters)

Reviewer #1: General comment: Please ask for English revision by a native speaker, the quality of English is not sufficient.

Abstract:

Number of caves should be mentioned.

Introduction:

Page1: Reference 1 is obsolete and looks like a Brazilian book which is hardly available in other countries. Consider to replace it by some current review or leave this general statement without any reference.

Page2: I think that nine references cited for a single simple statement are too much. Is there any review to be referred instead?

15 references mentioned (numbers 2-16) deal with sand fly fauna in caves and this fact puts in doubt the following statement "However, despite all these investigation, little is known of the patterns of distribution of sand flies in caves..." Try to explain more clearly what is the novelty of your work.

Materials and Methods

Explain why different numbers of traps were used in different cages and what was the distance between traps within one cage. All these details may affect catches per trap.

Add which keys were used for morphological identification of sand flies.

Are there any males or rest of female bodies available in ethanol for eventual molecular studies (confirmation of species identification or population studies) in the future?

Results

Did you trap any males with noninverted genitalia or do you have any other evidence that caves serve as breeding sites for sand flies?

In Table 2, explain what is T. Do you mean "n"?

I am missing the information about sand fly "density", for example the sand fly number per night and trap.

Do you have any data about temperature and humidity in caves versus their surroundings?

Discussion

You are frequently using term "condominium". Do you mean village or single house?

I suggest to improve/change discussion why caves are important for "the maintenance of their sand fly communities". Could you specify if these caves are serving as resting sites, breeding sites, and/or what else?

Reviewer #2: The manuscript entitled “Similarity of species composition of sand flies (Diptera: Psychodidae) in caves of Quadrilátero Ferrífero, state Minas Gerais, Brasil“ described an interesting study concerning the sand fly fauna in caves and its surroundings. Several major points need to be addressed before publishing this work, and therefore, this manuscript cannot be published in its actual form.

The authors reported a similarity of sand fly communities among the entrance, interior, and surroundings environments of each sampled cave at places, MS and PESRM. My major concern with this study is that only the analysis was based on a total of 375 sand flies caught during a whole year period. With such low number, no conclusion can be drawn. By using other traps, the authors may increase the number of sand flies needed for an objective statistical analysis. On the other hand, the authors should compare the similarity on a monthly basis and NOT during the whole period because it is well known that phenology differ among species. In addition, the authors should report Leishmania infection in both sites.

**********

6. PLOS authors have the option to publish the peer review history of their article (what does this mean?). If published, this will include your full peer review and any attached files.

If you choose “no”, your identity will remain anonymous but your review may still be made public.

**Do you want your identity to be public for this peer review?** For information about this choice, including consent withdrawal, please see our Privacy Policy.

Reviewer #1: No

Reviewer #2: No

[NOTE: If reviewer comments were submitted as an attachment file, they will be attached to this email and accessible via the submission site. Please log into your account, locate the manuscript record, and check for the action link "View Attachments". If this link does not appear, there are no attachment files to be viewed.]

While revising your submission, please upload your figure files to the Preflight Analysis and Conversion Engine (PACE) digital diagnostic tool, https://pacev2.apexcovantage.com/. PACE helps ensure that figures meet PLOS requirements. To use PACE, you must first register as a user. Registration is free. Then, login and navigate to the UPLOAD tab, where you will find detailed instructions on how to use the tool. If you encounter any issues or have any questions when using PACE, please email us at figures@plos.org. Please note that Supporting Information files do not need this step.

# Author comment: Author response to Decision Letter 0

**DOI:** 10.1371/journal.pone.0220268.r002

9 Oct 2019

Dear editor,

Below are the responses to the reviewers' comments.

We are available for clarification.

Reviewer #1: General comment: Please ask for English revision by a native speaker, the quality of English is not sufficient. 

Answer: The English was revision by a native speaker.

Abstract: Number of caves should be mentioned. 

Answer: The number of caves was mentioned.

Introduction: Page1: Reference 1 is obsolete and looks like a Brazilian book which is hardly available in other countries. Consider to replace it by some current review or leave this general statement without any reference.

Answer: The reference 1 was taken from the page 1 of the introduction.

Page2: I think that nine references cited for a single simple statement are too much. Is there any review to be referred instead?

Answer: Only one reference was left for each environment studied, urban, rural, wild and cave (1, 2, 3, 4). And there is no any review to be referred instead.

15 references mentioned (numbers 2-16) deal with sand fly fauna in caves and this fact puts in doubt the following statement "However, despite all these investigation, little is known of the patterns of distribution of sand flies in caves..." Try to explain more clearly what is the novelty of your work.

Answer: It appears that many projects have been carried out in Brazil's caves, but given the size of the country and the number of existing caves, this number becomes small. In addition, this is the first systematic work carried out in Brazil's iron caves.

Materials and Methods: Explain why different numbers of traps were used in different cages and what was the distance between traps within one cage. All these details may affect catches per trap.

Answer: The caves sizes were different. Was installed the maximum possible number of traps that fit within each cave, however were made some modifications in text to improve understanding. The distance between traps within one cave is in the text “For all caves and their surroundings sampled, the first trap inside a cave was at its entrance and the other was set every 10 m further into the cave. In the surroundings, the first trap was set in the forest 10 m from the cave entrance and the others were set every 10 m further away from the cave into the forest.” Modifications were done in the topics Collection of sand flies, Sand fly identification and Statistical analysis of the Materials and Methods.

Add which keys were used for morphological identification of sand flies.

Answer: This information was added to the text with some modifications.

Are there any males or rest of female bodies available in ethanol for eventual molecular studies (confirmation of species identification or population studies) in the future?

Answer: No. The information in line 152-153 “and females were placed in test tubes containing 6% DMSO for future study of Leishmania DNA detection” was taken from the text due to a misunderstanding, since the study was only ecological. In the future we will do studies of this kind.

Results: Did you trap any males with noninverted genitalia or do you have any other evidence that caves serve as breeding sites for sand flies?

Answer: Was not found any males with noninverted genitalia, as it is very difficult to collect sand flies at this stage of development. An evidence that caves serve as breeding sites for sand flies was the records of presence of troglophils species. 

In Table 2, explain what is T. Do you mean "n"?

Answer: Was explain in table caption 2 what T means. We also added other information to table 2. 

I am missing the information about sand fly "density", for example the sand fly number per night and trap.

Answer: The number of sand flies collected was low, a fact common in this type of environment (Quadrilátero Ferrífero), so we prefer not to do these analyzes of sand flies collected by trap and per night.

Do you have any data about temperature and humidity in caves versus their surroundings?

Answer: We do not have this information. At the beginning of the project our idea was to measure humidity and temperature, however we had several problems with the devices, from thermometer breakage to loss (probable theft), so we did not measure the climate variables.

Discussion

You are frequently using term "condominium". Do you mean village or single house?

Answer: Condominiums are houses in a periurban environment, surrounded by walls. The entry of people is controlled. It is common in the metropolitan region of Belo Horizonte and presents preserved vegetation and wildlife present near the residents.

I suggest to improve/change discussion why caves are important for "the maintenance of their sand fly communities". Could you specify if these caves are serving as resting sites, breeding sites, and/or what else?

Answer: Information was added in this part of the discussion to improve understanding of the conclusions reached.

Reviewer #2: The manuscript entitled “Similarity of species composition of sand flies (Diptera: Psychodidae) in caves of Quadrilátero Ferrífero, state Minas Gerais, Brasil“ described an interesting study concerning the sand fly fauna in caves and its surroundings. Several major points need to be addressed before publishing this work, and therefore, this manuscript cannot be published in its actual form.

The authors reported a similarity of sand fly communities among the entrance, interior, and surroundings environments of each sampled cave at places, MS and PESRM. My major concern with this study is that only the analysis was based on a total of 375 sand flies caught during a whole year period. With such low number, no conclusion can be drawn. By using other traps, the authors may increase the number of sand flies needed for an objective statistical analysis. On the other hand, the authors should compare the similarity on a monthly basis and NOT during the whole period because it is well known that phenology differ among species. In addition, the authors should report Leishmania infection in both sites.

Answer: The low number of sand flies collected is a characteristic of the studied region (Quadrilátero Ferrífero) and is not a problem of the methodology used. The fact that it has few sand flies compared to, for example, a limestone cave should not be an impediment to studying it. Information was added in the discussion topic for a better understanding of the overall conclusions obtained as well as for the clarification of the statistical analysis used to arrive at an appropriate and reliable conclusion. The comparison of sand flies fauna on a monthly basis will not be possible, since there have been many months with only one species or few species of sand flies collected, which made it impossible to compare the similarity monthly. The NMDS analysis can be considered useful in trying to find out if there are patterns in the ecological data, which was the purpose of the study. Thus, we believe that analyzing the similarity of the phlebotomine community by caves and their surroundings during the entire collection period is the best way for what was proposed in the present study, since the NMDS analysis was to investigate the composition of sand fly species based on the presence and absence of species inside and outside the caves regardless of the sampling period, in other words, was an analysis to evaluate the spatial distribution of the community and not temporal distribution. The Leishmania detection study was not performed in the studied sites, because we had methodological problems when performing sample extraction which made this part of the study impossible.

Attachment Submitted filename: Response to Reviewers.doc Click here for additional data file.

# Peer review: Decision Letter 1

**DOI:** 10.1371/journal.pone.0220268.r003

**Authors:** Albert Schriefer

30 Dec 2019

PONE-D-19-18993R1

Species composition of sand flies (Diptera: Psychodidae) in caves of Quadrilátero Ferrífero, state of Minas Gerais, Brazil

PLOS ONE

Dear Dr. Andrade Filho,

Thank you for submitting your manuscript to PLOS ONE. After careful consideration, we feel that it has merit but does not fully meet PLOS ONE’s publication criteria as it currently stands. Therefore, we invite you to submit a revised version of the manuscript that addresses the points raised during the review process.

Given the conflicting judgements in the original reviews, two novel reviewers were invited. Language has been thoroughly improved, but all four reviewers raised points that still need your attention. Please, be generous in incorporating the suggestions. Below, I also made some minor comments in order to further contribute with the report. When responding to the reviewers, make sure to clearly state the line were the comment / suggestion was addressed / incorporated in the revised manuscript's text.

We would appreciate receiving your revised manuscript by Feb 13 2020 11:59PM. When you are ready to submit your revision, log on to https://www.editorialmanager.com/pone/ and select the 'Submissions Needing Revision' folder to locate your manuscript file.

If you would like to make changes to your financial disclosure, please include your updated statement in your cover letter.

To enhance the reproducibility of your results, we recommend that if applicable you deposit your laboratory protocols in protocols.io, where a protocol can be assigned its own identifier (DOI) such that it can be cited independently in the future. For instructions see: http://journals.plos.org/plosone/s/submission-guidelines#loc-laboratory-protocols

Please include the following items when submitting your revised manuscript:

- A rebuttal letter that responds to each point raised by the academic editor and reviewer(s). This letter should be uploaded as separate file and labeled 'Response to Reviewers'.
- A marked-up copy of your manuscript that highlights changes made to the original version. This file should be uploaded as separate file and labeled 'Revised Manuscript with Track Changes'.
- An unmarked version of your revised paper without tracked changes. This file should be uploaded as separate file and labeled 'Manuscript'.

Please note while forming your response, if your article is accepted, you may have the opportunity to make the peer review history publicly available. The record will include editor decision letters (with reviews) and your responses to reviewer comments. If eligible, we will contact you to opt in or out.

We look forward to receiving your revised manuscript.

Kind regards,

Albert Schriefer, M.D., Ph.D.

Academic Editor

PLOS ONE

Additional Editor Comments (if provided):

1- Add one or two paragraphs in discussion dealing with the limitations faced by the study, and to what extent they may have influenced conclusions. Please, make sure to include the following points raised by more than one reviewer, but also expand to other possible limitations: (1) the analyses were based on a limited sample; (2) seasonality was not approached; (3) the study should have encompassed more than a single year.

2- I also suggest adding a paragraph to discussion indicating the existence and overall findings of similar studies conducted in other New World and Old World countries, in order to improve contextualization of the current study.

3- State in discussion which of the species described in Table 2 were implicated in transmission of Leishmania parasites to human beings, and which were not.

4- Discussion, line 273. Please correct Leismania infatum with Leismania infantum.

[Note: HTML markup is below. Please do not edit.]

Reviewers' comments:

Reviewer's Responses to Questions

**Comments to the Author**

1. If the authors have adequately addressed your comments raised in a previous round of review and you feel that this manuscript is now acceptable for publication, you may indicate that here to bypass the “Comments to the Author” section, enter your conflict of interest statement in the “Confidential to Editor” section, and submit your "Accept" recommendation.

Reviewer #1: (No Response)

Reviewer #2: (No Response)

Reviewer #3: (No Response)

Reviewer #4: All comments have been addressed

**********

2. Is the manuscript technically sound, and do the data support the conclusions?

The manuscript must describe a technically sound piece of scientific research with data that supports the conclusions. Experiments must have been conducted rigorously, with appropriate controls, replication, and sample sizes. The conclusions must be drawn appropriately based on the data presented. 

Reviewer #1: Yes

Reviewer #2: Yes

Reviewer #3: Partly

Reviewer #4: Partly

**********

3. Has the statistical analysis been performed appropriately and rigorously? 

Reviewer #1: I Don't Know

Reviewer #2: Yes

Reviewer #3: Yes

Reviewer #4: I Don't Know

**********

4. Have the authors made all data underlying the findings in their manuscript fully available?

The PLOS Data policy requires authors to make all data underlying the findings described in their manuscript fully available without restriction, with rare exception (please refer to the Data Availability Statement in the manuscript PDF file). The data should be provided as part of the manuscript or its supporting information, or deposited to a public repository. For example, in addition to summary statistics, the data points behind means, medians and variance measures should be available. If there are restrictions on publicly sharing data—e.g. participant privacy or use of data from a third party—those must be specified.

Reviewer #1: Yes

Reviewer #2: Yes

Reviewer #3: Yes

Reviewer #4: (No Response)

**********

5. Is the manuscript presented in an intelligible fashion and written in standard English?

PLOS ONE does not copyedit accepted manuscripts, so the language in submitted articles must be clear, correct, and unambiguous. Any typographical or grammatical errors should be corrected at revision, so please note any specific errors here.

Reviewer #1: Yes

Reviewer #2: Yes

Reviewer #3: No

Reviewer #4: Yes

**********

6. Review Comments to the Author

Please use the space provided to explain your answers to the questions above. You may also include additional comments for the author, including concerns about dual publication, research ethics, or publication ethics. (Please upload your review as an attachment if it exceeds 20,000 characters)

Reviewer #1: Some comments were handled satisfactorily, some were answered in the reply to reviewer only.

It is necessary to reflect at least two following comments also in the text of the manuscript.

: Explain in Discussion why the sand fly number per night and trap is not given.

: The term "condominium" needs to be explained in the text, please understand that you are writing also for non-Brazilian audience.

Finally, it is good that you indicated which taxonomy (Galati, 2003) was used to classify sand fly species, however, I am still missing the information about keys used for morphological identification.

Reviewer #2: The revised manuscript entitled “Species composition of sand flies (Diptera: Psychodidae) in caves of Quadrilátero Ferrífero, state Minas Gerais, Brasil“ described a study concerning the sand fly fauna in caves and its surroundings. In this revision, authors did not address major points raised previously. In addition, arguments advanced by authors were not convincing.

My major concern with this study is that the analysis was based only on a total of 375 sand flies caught during a whole year period. With such low number, no conclusion can be drawn. To be accepted, this entomological survey should be performed over a 2-year period.

In conclusion, this manuscript in its revised form should not be accepted for publication

Reviewer #3: This study aimed to address the phlebotomine fauna in caves in a mining region of Minas Gerais state Brazil. The study is very preliminary/descriptive and did not ascertain sandfly infection by Leishmania. This is of importance since several hosts can also use the caves and hence, be potential blood sources for the vectors. I think this manuscript is more suitable for a short communication elsewhere. Although the authors claim that the revision was made by a native English speaker, I myself found several spelling and grammar mistakes in the text. I will indicate some. I therefor recommend rejection of the manuscript in its current form.

Abstract

Lines 16-18 - "Caves, though seemingly inhospitable due to the lack of light in areas furthest from the

entrance and the low availability and variety of resources, can harbor many species of

vertebrates and invertebrates."

Although I understand what the authors meant the sentence would be much easier to understand as follows:

Caves are extreme and inhospitable environments that can harbour several species of vertebrate and invertebrate animals.

Lines 20-22 - The authors shouls state that one cave in is MS and the other three in PNSRL, by reading the abstract we have the idea that four caves in each area.

Introduction

Lines 44-47 - sentences identical to abstract. The authors should avoid this type of thing.

Lines 53-54 - "differences in the period of activity or in its diversity among several caves of the same region have been shown". I would suggest as follows: differences in activity periods and diversity among several caves of the same region have been reported.

Line 62 - "Given the above" - please remove this and start the sentence with "Few studies ...

Lines 66-69 - Very long and confusing sentence, please consider shortening and reviewing the English.

Line 69 - "then" is unnecessary.

Line 70 - "to use ordination to structure local communities along an environmental gradient" - although I have worked with sandflies collection, I have no idea what the authors meant with this sentence. Considering revising the text.

Lines 79-83 - The authors claim that knowing the vector could help to avoid transmition of Leishmaniasis to humans. However, they did not perform PCR for parasite detection. This is the main weakness of this manuscript.

Line 179 - "whereas in the most abundant" replace for whereas the most abundant.

Lines 182 a 185 - consider english revision.

Suggestion: In Cave RM39, a total of 190 individuals were collected: 48 inside and 142 outside. Ev. tupynambai (76.1%) was the most abundant species inside whereas Br. troglodytes (81.2%) was the dominant species outside.

Lines 204-205 - "as was observed for" - this sentence should be completely rewritten.

Line 240, 270 - longipalpis not Longipalpis;

Line 273 - infantum not infatum;

Line 273 - although L. longipalpis is an incriminated vector for VL, in Lapinha Cave there is also this species and no transmission of VL in that area. The authors cannot conclude anything about the status of L. longipalpis in this cave without at least performing PCR to detect L. infantum.

Reviewer #4: Dear Editor,

Below is my summary opinion regard to the article sent to me for reviewing.

The authors reported a similarity of sand fly communities among the entrance, interior, and surroundings environments of each sampled cave located at, MS and PESRM. However, the insuficiente information to reach any firm conclusion due to a lack of season records of the collected species may weaken the reality of species composition or patterns of distribution. This means that the overall analysis obtained, although assured by the statistical tests, may not represent a similarity of sand fly communities in specific periods at places MS and PESRM.

The authors properly exhibited the study area, including trap distribution mode, sample collection and period. On the other hand, they did not include data on environmental descriptors (vegetation, soil type), temperature and humidity at the traps attachment points in caves and its surroundings, impairing the discussion on stratifications of the sandflies.

The manuscript entitled “Species composition of sand flies (Diptera: Psychodidae) in caves of Quadrilátero Ferrífero, state Minas Gerais, Brasil“ described an interesting study of sandflies fauna, including the finding of Evandromyia tupynambai notably predominating inside PESRM caves.

In my opinion, the presentation of the paper could be conducted more as a survey of sand flies fauna in the Quadrilátero Ferrífero, state of Minas Gerais, Brazil than as a study that responds patterns of distribution of sand fly communities among the entrance, interior, and surroundings environments of each sampled cave at places, MS and PESRM.

**********

7. PLOS authors have the option to publish the peer review history of their article (what does this mean?). If published, this will include your full peer review and any attached files.

If you choose “no”, your identity will remain anonymous but your review may still be made public.

**Do you want your identity to be public for this peer review?** For information about this choice, including consent withdrawal, please see our Privacy Policy.

Reviewer #1: No

Reviewer #2: No

Reviewer #3: No

Reviewer #4: No

[NOTE: If reviewer comments were submitted as an attachment file, they will be attached to this email and accessible via the submission site. Please log into your account, locate the manuscript record, and check for the action link "View Attachments". If this link does not appear, there are no attachment files to be viewed.]

While revising your submission, please upload your figure files to the Preflight Analysis and Conversion Engine (PACE) digital diagnostic tool, https://pacev2.apexcovantage.com/. PACE helps ensure that figures meet PLOS requirements. To use PACE, you must first register as a user. Registration is free. Then, login and navigate to the UPLOAD tab, where you will find detailed instructions on how to use the tool. If you encounter any issues or have any questions when using PACE, please email us at figures@plos.org. Please note that Supporting Information files do not need this step.

# Author comment: Author response to Decision Letter 1

**DOI:** 10.1371/journal.pone.0220268.r004

10 Feb 2020

Dear editor,

Below are the responses to the editor and reviewers' comments.

We are available for clarification.

Responses to editor comments:

1- Add one or two paragraphs in discussion dealing with the limitations faced by the study, and to what extent they may have influenced conclusions. Please, make sure to include the following points raised by more than one reviewer, but also expand to other possible limitations: (1) the analyses were based on a limited sample; (2) seasonality was not approached; (3) the study should have encompassed more than a single year.

Answer: All requested information has been added to the text from line 306-337.

2- I also suggest adding a paragraph to discussion indicating the existence and overall findings of similar studies conducted in other New World and Old World countries, in order to improve contextualization of the current study.

Answer: We have included a paragraph about some studies of sand flies in caves. If the reviewer deems it necessary, we can include other data and include the necessary references.

3- State in discussion which of the species described in Table 2 were implicated in transmission of Leishmania parasites to human beings, and which were not.

Answer: This information was added to the text at line 262, 264-265.

4- Discussion, line 273. Please correct Leismania infatum with Leismania infantum.

Answer: The name of this species has been corrected in line 292.

Responses to reviewers comments:

Reviewer #1:

Explain in Discussion why the sand fly number per night and trap is not given.

Answer: This information was added from line 327-329.

The term "condominium" needs to be explained in the text, please understand that you are writing also for non-Brazilian audience. 

Answer: To facilitate understanding, the term residential condominium was replaced by residential area in a periurban environment in line 252.

Finally, it is good that you indicated which taxonomy (Galati, 2003) was used to classify sand fly species, however, I am still missing the information about keys used for morphological identification.

Answer: This information was added in line 160.

Reviewer #2:

The revised manuscript entitled “Species composition of sand flies (Diptera: Psychodidae) in caves of Quadrilátero Ferrífero, state Minas Gerais, Brasil“ described a study concerning the sand fly fauna in caves and its surroundings. In this revision, authors did not address major points raised previously. In addition, arguments advanced by authors were not convincing.

Answer: Information regarding these questions has been added from line 306-337 for better clarification regarding the proposed study.

My major concern with this study is that the analysis was based only on a total of 375 sand flies caught during a whole year period. With such low number, no conclusion can be drawn. To be accepted, this entomological survey should be performed over a 2-year period.

Answer: We made some clarifications regarding these questions from line 306-337.

Reviewer #3:

This study aimed to address the phlebotomine fauna in caves in a mining region of Minas Gerais state Brazil. The study is very preliminary/descriptive and did not ascertain sandfly infection by Leishmania. This is of importance since several hosts can also use the caves and hence, be potential blood sources for the vectors. I think this manuscript is more suitable for a short communication elsewhere. Although the authors claim that the revision was made by a native English speaker, I myself found several spelling and grammar mistakes in the text. I will indicate some. I therefor recommend rejection of the manuscript in its current form.

Answer: Modifications were made throughout the entire text of the article, with emphasis on line 306-337, in order to clarify all these questions. In addition, English has been revised. 

Line: 16-18 - "Caves, though seemingly inhospitable due to the lack of light in areas furthest from the entrance and the low availability and variety of resources, can harbor many species of vertebrates and invertebrates."

Answer: Subject accepts in line 16-19.

Lines 20-22 - The authors shouls state that one cave in is MS and the other three in PNSRL, by reading the abstract we have the idea that four caves in each area.

Answer: The line 22 has been rewritten in this part of the abstract to improve understanding.

Lines 44-47 - sentences identical to abstract. The authors should avoid this type of thing.

Answer: The Line 22 has been rewritten, so there are no more sentences identical to abstract.

Lines 53-54 - "differences in the period of activity or in its diversity among several caves of the same region have been shown". I would suggest as follows: differences in activity periods and diversity among several caves of the same region have been reported.

Answer: Subject accepts in line 54-56.

Line 62 - "Given the above" - please remove this and start the sentence with "Few studies ...

Answer: This whole paragraph, from line 64-76, has been replaced by the sentence from line 77-88. The English has also been revised.

Lines 66-69 - Very long and confusing sentence, please consider shortening and reviewing the English.

Answer: This whole paragraph, from line 64-76, has been replaced by the sentence from line 77-88. The English has also been revised.

Line 69 - "then" is unnecessary.

Answer: This whole paragraph, from line 64-76, has been replaced by the sentence from line 77-88. The English has also been revised.

Line 70 - "to use ordination to structure local communities along an environmental gradient" - although I have worked with sandflies collection, I have no idea what the authors meant with this sentence. Considering revising the text.

Answer: This paragraph has been restructured from line 83-88 to better understanding the context. English has also been revised.

Lines 79-83 - The authors claim that knowing the vector could help to avoid transmition of Leishmaniasis to humans. However, they did not perform PCR for parasite detection. This is the main weakness of this manuscript.

Answer: This paragraph has been removed from the line 93-97, because we consider it unnecessary for the study.

Line 179 - "whereas in the most abundant" replace for whereas the most abundant.

Answer: Sentence has been corrected in line 194.

Lines 182 a 185 - consider english revision.

Answer: The English was revision by a native speaker.

Suggestion: In Cave RM39, a total of 190 individuals were collected: 48 inside and 142 outside. Ev. tupynambai (76.1%) was the most abundant species inside whereas Br. troglodytes (81.2%) was the dominant species outside.

Answer: Suggestion was accepted from line 197-203.

Lines 204-205 - "as was observed for" - this sentence should be completely rewritten.

Answer: We agreed that this sentence was really misspelled with out of order affirmations, so this paragraph has been restructured from line 220-232.

Line 240, 270 - longipalpis not Longipalpis;

Answer: The name of this species has been corrected in line 259, 289.

Line 273 - infantum not infatum;

Answer: The name of this species has been corrected in line 292.

Line 273 - although L. longipalpis is an incriminated vector for VL, in Lapinha Cave there is also this species and no transmission of VL in that area. The authors cannot conclude anything about the status of L. longipalpis in this cave without at least performing PCR to detect L. infantum.

Answer: The reviewer is partly right. Lutzomyia longipalpis is known to be a species complex and probably with variable vector capacity between populations / species within the complex (see Casanova et al. 2015), for this reason we do not claim that Lu. longipalpis is the vector in the studied area, even though we do not know if there is Leishmania circulation in the studied environment. We only mention two species cited to Leishmania in other places

Regarding the population of Lu. longipalpis from Lapinha cave, we cannot say that there is no transmission of VL, as there are no systematic studies on the natural infection / detection of Leishmania DNA in that population. Several studies on experimental infection have already been carried out in colonies from the Lapinha cave (Secundino et al. 2012; Freitas et al 2012; Laurenti et al. 2009). A curious fact in relation to this area, is that one of the creators of the studies with Lu. longipalpis in this area, researcher Alberto Falcão, was infected with visceral leishmaniasis in Gruta da Lapinha in the 60s

Reviewer #4: 

The authors reported a similarity of sand fly communities among the entrance, interior, and surroundings environments of each sampled cave located at, MS and PESRM. However, the insuficiente information to reach any firm conclusion due to a lack of season records of the collected species may weaken the reality of species composition or patterns of distribution. This means that the overall analysis obtained, although assured by the statistical tests, may not represent a similarity of sand fly communities in specific periods at places MS and PESRM.

Answer: Modifications were made throughout the entire text of the article, with emphasis on line 306-337, in order to clarify all these questions.

The authors properly exhibited the study area, including trap distribution mode, sample collection and period. On the other hand, they did not include data on environmental descriptors (vegetation, soil type), temperature and humidity at the traps attachment points in caves and its surroundings, impairing the discussion on stratifications of the sandflies.

Answer: Modifications were made throughout the entire text of the article, with emphasis on line 306-337, in order to clarify all these questions. 

In my opinion, the presentation of the paper could be conducted more as a survey of sand flies fauna in the Quadrilátero Ferrífero, state of Minas Gerais, Brazil than as a study that responds patterns of distribution of sand fly communities among the entrance, interior, and surroundings environments of each sampled cave at places, MS and PESRM.

Answer: Modifications from line 89-93 were made to better understanding the proposed study.

Attachment Submitted filename: Response to Reviewers.doc Click here for additional data file.

# Editor report: Decision Letter 2

**DOI:** 10.1371/journal.pone.0220268.r005

**Authors:** Albert Schriefer

19 Feb 2020

PONE-D-19-18993R2

Species composition of sand flies (Diptera: Psychodidae) in caves of Quadrilátero Ferrífero, state of Minas Gerais, Brazil

PLOS ONE

Dear Dr. Andrade Filho,

Thank you for submitting your manuscript to PLOS ONE. After careful consideration, we feel that it has merit but does not fully meet PLOS ONE’s publication criteria as it currently stands. Therefore, we invite you to submit a revised version of the manuscript that addresses the points raised during the review process.

Please, before we can proceed with your manuscript, fully incorporate the few additional editor comments at the end of this letter.

We would appreciate receiving your revised manuscript by Apr 04 2020 11:59PM. When you are ready to submit your revision, log on to https://www.editorialmanager.com/pone/ and select the 'Submissions Needing Revision' folder to locate your manuscript file.

If you would like to make changes to your financial disclosure, please include your updated statement in your cover letter.

To enhance the reproducibility of your results, we recommend that if applicable you deposit your laboratory protocols in protocols.io, where a protocol can be assigned its own identifier (DOI) such that it can be cited independently in the future. For instructions see: http://journals.plos.org/plosone/s/submission-guidelines#loc-laboratory-protocols

Please include the following items when submitting your revised manuscript:

- A rebuttal letter that responds to each point raised by the academic editor and reviewer(s). This letter should be uploaded as separate file and labeled 'Response to Reviewers'.
- A marked-up copy of your manuscript that highlights changes made to the original version. This file should be uploaded as separate file and labeled 'Revised Manuscript with Track Changes'.
- An unmarked version of your revised paper without tracked changes. This file should be uploaded as separate file and labeled 'Manuscript'.

Please note while forming your response, if your article is accepted, you may have the opportunity to make the peer review history publicly available. The record will include editor decision letters (with reviews) and your responses to reviewer comments. If eligible, we will contact you to opt in or out.

We look forward to receiving your revised manuscript.

Kind regards,

Albert Schriefer, M.D., Ph.D.

Academic Editor

PLOS ONE

Additional Editor Comments (if provided):

1- As repeatedly requested by reviewer 1, please, completely disclose the missing the information about keys used for morphological identification.

2- Lines 64 to 66. Correct to "Therefore, no ecological studies have been carried out in ferruginous environments evaluating the environmental gradient of caves and its surrounding phebotomine communities.

3- Line 70. Correct from "structure" to "structured".

4- Line 293. add a 'to' in 'for example, to a limestone cave [1, 4]'.

[NOTE: If reviewer comments were submitted as an attachment file, they will be attached to this email and accessible via the submission site. Please log into your account, locate the manuscript record, and check for the action link "View Attachments". If this link does not appear, there are no attachment files to be viewed.]

While revising your submission, please upload your figure files to the Preflight Analysis and Conversion Engine (PACE) digital diagnostic tool, https://pacev2.apexcovantage.com/. PACE helps ensure that figures meet PLOS requirements. To use PACE, you must first register as a user. Registration is free. Then, login and navigate to the UPLOAD tab, where you will find detailed instructions on how to use the tool. If you encounter any issues or have any questions when using PACE, please email us at figures@plos.org. Please note that Supporting Information files do not need this step.

# Author comment: Author response to Decision Letter 2

**DOI:** 10.1371/journal.pone.0220268.r006

19 Feb 2020

Dear Academic Editor,

Below the answers to the questions

1- As repeatedly requested by reviewer 1, please, completely disclose the missing the information about keys used for morphological identification.

Answer: In the last version, the phrase on identification and classification of sandflies used in the article was modified (All sand flies were identified following the key and classification of Galati (2003) [30], lines 140 and 141. In case it is not clear we can change in order to facilitate the understanding of readers

2- Lines 64 to 66. Correct to "Therefore, no ecological studies have been carried out in ferruginous environments evaluating the environmental gradient of caves and its surrounding phlebotomine communities.

Answer: Change accepted

3- Line 70. Correct from "structure" to "structured".

Answer: Change accepted

4- Line 293. add a 'to' in 'for example, to a limestone cave [1, 4]'.

Answer: Change accepted

Attachment Submitted filename: Rebuttal Letter.doc Click here for additional data file.

# Editor report: Decision Letter 3

**DOI:** 10.1371/journal.pone.0220268.r007

**Authors:** Albert Schriefer

20 Feb 2020

Species composition of sand flies (Diptera: Psychodidae) in caves of Quadrilátero Ferrífero, state of Minas Gerais, Brazil

PONE-D-19-18993R3

Dear Dr. Andrade Filho,

We are pleased to inform you that your manuscript has been judged scientifically suitable for publication and will be formally accepted for publication once it complies with all outstanding technical requirements.

Within one week, you will receive an e-mail containing information on the amendments required prior to publication. When all required modifications have been addressed, you will receive a formal acceptance letter and your manuscript will proceed to our production department and be scheduled for publication.

Shortly after the formal acceptance letter is sent, an invoice for payment will follow. To ensure an efficient production and billing process, please log into Editorial Manager at https://www.editorialmanager.com/pone/, click the "Update My Information" link at the top of the page, and update your user information. If you have any billing related questions, please contact our Author Billing department directly at authorbilling@plos.org.

If your institution or institutions have a press office, please notify them about your upcoming paper to enable them to help maximize its impact. If they will be preparing press materials for this manuscript, you must inform our press team as soon as possible and no later than 48 hours after receiving the formal acceptance. Your manuscript will remain under strict press embargo until 2 pm Eastern Time on the date of publication. For more information, please contact onepress@plos.org.

With kind regards,

Albert Schriefer, M.D., Ph.D.

Academic Editor

PLOS ONE

Additional Editor Comments (optional):

Reviewers' comments:

# Editor report: Acceptance letter

**DOI:** 10.1371/journal.pone.0220268.r008

**Authors:** Albert Schriefer

24 Feb 2020

PONE-D-19-18993R3 

Species composition of sand flies (Diptera: Psychodidae) in caves of Quadrilátero Ferrífero, state of Minas Gerais, Brazil 

Dear Dr. Andrade Filho:

I am pleased to inform you that your manuscript has been deemed suitable for publication in PLOS ONE. Congratulations! Your manuscript is now with our production department. 

If your institution or institutions have a press office, please notify them about your upcoming paper at this point, to enable them to help maximize its impact. If they will be preparing press materials for this manuscript, please inform our press team within the next 48 hours. Your manuscript will remain under strict press embargo until 2 pm Eastern Time on the date of publication. For more information please contact onepress@plos.org.

For any other questions or concerns, please email plosone@plos.org. 

Thank you for submitting your work to PLOS ONE.

With kind regards,

PLOS ONE Editorial Office Staff

on behalf of

Dr. Albert Schriefer 

Academic Editor

PLOS ONE
